# The Impact of 10 Unmalted Alternative Adjuncts on Wort Characteristics

**DOI:** 10.3390/foods12234206

**Published:** 2023-11-22

**Authors:** David Laureys, Jeroen Baillière, Pieter Vermeir, Dana Vanderputten, Jessika De Clippeleer

**Affiliations:** 1 Innovation Centre for Brewing & Fermentation (IBF), Department of Biotechnology, Faculty of Bioscience Engineering, Ghent University, Valentin Vaerwyckweg 1, 9000 Ghent, Belgium; david.laureys@ugent.be (D.L.);; 2 Laboratory of Chemical Analysis (LCA), Department of Green Chemistry and Technology, Faculty of Bioscience Engineering, Ghent University, Valentin Vaerwyckweg 1, 9000 Ghent, Belgium; pieter.vermeir@ugent.be; 3 Innovation Centre for Brewing & Fermentation (IBF), AgroFoodNature, HOGENT University of Applied Sciences and Arts, Valentin Vaerwyckweg 1, 9000 Ghent, Belgium; dana.vanderputten@hogent.be

**Keywords:** adjunct, pseudocereal, cereal, mashing, wort, brewing, extract

## Abstract

Consumers are more than ever in search of novel and exciting beer choices, and brewers are, therefore, continuously experimenting to adapt their product portfolio. One interesting way to naturally incorporate novel flavors and tastes is by using alternative adjuncts, but this is not always an easy and straightforward process. In this study, a 40% unmalted alternative adjunct (einkorn, emmer, spelt, khorasan, quinoa, amaranth, buckwheat, sorghum, teff, and tritordeum) or reference (barley malt, unmalted barley, and unmalted wheat) was added to 60% barley malt, after which three different laboratory mashing processes (Congress mash, Congress mash with pre-gelatinization of the adjunct, and Evans mash) were performed, and their behavior during mashing and the resulting wort characteristics were investigated in detail. Overall, the extraction process of all 10 unmalted alternative adjuncts was not complete for all three laboratory mashing processes, whereby Congress mashing resulted in the highest extract and fastest filtration, whereas Evans mashing resulted in the lowest extract and slowest filtration. Pre-gelatinization of the unmalted was generally only beneficial for adjuncts with high onset starch gelatinization temperatures. This process also inactivated endogenous enzymes in the unmalted adjuncts, which had an adverse effect on the mashing process.

## 1. Introduction

One of the most important trends in beer consumption during the last years is the changing preference from quantity to quality. Consumers are drinking less in total volumes but increasingly ask for high-quality specialty beers and novel experiences [1,2], as consumers now link the beer they drink to their identity [3]. In order to remain competitive in this continuously evolving market, brewers have been experimenting with many parameters of the beer production process, such as alternative mashing processes, the timing of hop addition, different hop extracts, novel yeast strains, the addition of herbs or fruits, and barrel aging and wood lagering [4,5]. Using alternative adjuncts also offers a natural way to incorporate novel flavors in beer, and a limited number of unmalted adjuncts (such as rice, maize, wheat, oats, and rye) are commonly used in the brewing industry [6,7,8,9]. However, many more alternative cereals and pseudocereals are available for which the brewing potential is currently unknown, indicating that the use of alternative unmalted adjuncts for introducing novel flavors and experiences in beer has not yet reached its full potential [8].

The use of unmalted adjuncts may also contribute to make the brewing process more sustainable by omitting the energy-intensive malting and kilning processes, which are also known to introduce certain undesirable compounds (such as aldehydes) into the pitching wort [10]. Additionally, some of these alternative cereals and pseudocereals are especially resistant to drought (for example, tritordeum) or disease (for example, einkorn) or can be cultivated under marginal conditions with low fertilizer input (for example, einkorn and emmer) [11,12]. When climatic conditions in certain regions become unfavorable for the cultivation of barley, the brewing industry might search for alternative crops with promising brewing potential. Lastly, some alternative cereals and pseudocereals are especially rich in micro-nutrients (for example, quinoa, amaranth, buckwheat, and teff) that may contribute to human nutrition [13], and some alternative cereals and pseudocereals are naturally low in gluten (for example, tritordeum) or gluten-free (for example, quinoa, amaranth, buckwheat, sorghum, and teff) and may be used for the production of gluten-free beers [14].

In previous research, 10 unmalted alternative cereals and pseudocereals were selected, and their characteristics relevant to the brewing process were compared with each other and with the references of barley malt and unmalted barley and wheat [6]. The selected alternative adjuncts encompassed ancient wheat varieties (einkorn, emmer, spelt, and khorasan), pseudocereals (quinoa, amaranth, and buckwheat), and in western markets, less widely used cereals (sorghum, teff, and tritordeum). Based on these characteristics, some predictions could be made about their suitability for the brewing process. However, a mashing process is the result of an intricate interplay between many factors (such as physical and physicochemical properties of the raw materials and their chemical and enzymatic composition), so performing small-scale mashing processes is indispensable to assess their real brewing potential. Traditionally, a Congress mashing procedure is performed, but recently, an alternative Evans mashing procedure was proposed to be more representative of the industrial processes [7,15].

The aims of this study were thus to assess the brewing potential of the aforementioned 10 alternative unmalted adjuncts (einkorn, emmer, spelt, khorasan, quinoa, amaranth, buckwheat, sorghum, teff, and tritordeum) relative to the aforementioned references (barley malt, and unmalted barley and wheat) by performing three different laboratory-scale mashing processes (Congress mash, Congress mash with pre-gelatinization of the adjunct, and the Evans mash) with 60% barley malt and 40% adjunct, describing the mashing and filtration processes and the resulting wort characteristics in detail. This should allow brewers to rationally select a certain uncommon adjunct for product innovation and diversification, facilitate its implementation in an actual brewing process, and indicate promising crops as alternatives for barley.

## 2. Materials and Methods

### 2.1. Experiments

Congress mashing, Congress mashing with pre-gelatinization, and Evans mashing procedures were performed in triplicate in laboratory mashing baths with 60% barley malt and 40% of each of the alternative adjuncts. These mashing procedures were also performed in triplicate for 100% barley malt, with 60% barley malt and 40% unmalted barley, or 40% unmalted wheat (as references). To minimize the influence of the different adjuncts on the mash pH, 100 mL of distilled water was substituted by 100 mL of 0.1 M acetate buffer at pH 5.2, as described before [7]. During each mashing process, the time to saccharification was measured, and after each mashing process, the filtration speed was assessed, and the extracted content, pH, wort color, free amino nitrogen, and sugar composition of the resulting wort were determined. Additionally, the beta-glucan content, polyphenol content, soluble nitrogen, and wort viscosity were also assessed after Congress mashing.

### 2.2. Malt and Adjuncts

Commercially available barley malt (Albert Maltings, Puurs-Sint-Amands, Belgium), barley (Albert Maltings, Puurs-Sint-Amands, Belgium), wheat (HVB-IMTC, Oudenaarde, Belgium), einkorn (Boerderij Bijlsma, Nieuw-Vennep, The Netherlands), emmer (Boerderij Bijlsma, Nieuw-Vennep, The Netherlands), spelt (Kollenberger Spelt, Geleen, The Netherlands), khorasan (Kamut Enterprises of Europe, Oudenaarde, Belgium), quinoa (AgriPollet, Zwevegem, Belgium), amaranth (Mill & Mix, Zedelgem, The Netherlands), buckwheat (Mill & Mix, Zedelgem, The Netherlands), sorghum (Maatschap De Milliano-Meijer, Oostburg, The Netherlands), teff (Millets Place, Gasselte, The Netherlands), and tritordeum (Agrasys, Barcelona, Spain) were obtained and characterized in detail, as described before [6]. Barley malt, barley, einkorn, and emmer were used in the husked form, while wheat, spelt, khorasan, quinoa, amaranth, buckwheat, sorghum, teff, and tritordeum were used in their dehusked form, as they were commercially available as such.

### 2.3. Mashing Procedures

The Congress mashing procedure was based on the European Brewery Convention (EBC) method 4.5. Briefly, 30 g of barley malt (60%) and 20 g of adjunct (40%) were milled at 0.2 mm with a laboratory disc mill (Bühler, Uzwil, Switzerland) and added to 100 mL of a 0.1 M acetate buffer at pH 5.2 and 100 mL of distilled water to obtain a dilution of 1:4. This mash was placed in a LB Electronic mashing bath (Lochner Labor und Technik GmbH, Berching, Germany) with a continuous stirring speed of 200 rpm. The temperature was kept at 45 °C for 30 min, followed by a temperature increase of 1 °C/min until 70 °C, after which 100 mL of distilled water was added to obtain a dilution of 1:6. The mash was kept at 70 °C for 60 min, after which the mash was cooled to 20 °C and distilled water was added until 450 g to obtain a final mash dilution of 1:8.

For the Congress mashing procedure with pre-gelatinization, 20 g of the adjunct (40%) was milled at 0.2 mm and added to 100 mL of a 0.1 M acetate buffer and heated at 95 °C for 20 min, after which 100 mL of cold distilled water was added and the mash was further cooled to 45 °C whereafter 30 g of barley malt milled at 0.2 mm was added, and the mashing process was continued as described above.

The Evans mashing procedure was based on re-evaluating the small-scale Congress mash protocol [15]. Briefly, 30 g of barley malt (60%) and 20 g of the adjunct (40%) were milled at 0.7 mm with a laboratory disc mill (Bühler, Uzwil, Switzerland) and added to 100 mL of a 0.1 M acetate buffer at pH 5.2 and 50 mL of 0.9 mM CaCl_2_ in distilled water to obtain a dilution of 1:3. The temperature was kept at 65 °C for 50 min, followed by a temperature increase of 2.5 °C/min until 74 °C. The mash was kept at 74 °C for 10 min, after which 100 mL of distilled water was added, and the mash was cooled to 20 °C, whereafter distilled water was added until 450 g to obtain a final mash dilution of 1:8.

### 2.4. Time to Saccharification

The time to complete saccharification was determined as described in the EBC method 4.5.1. Briefly, a drop of iodine solution was added to a drop of mash, whereby saccharification resulted in a yellow color instead of a dark blue color. For the Congress mashing procedures, a saccharification test was performed every 10 min after the temperature reached 70 °C, and for the Evans mashing procedure, a saccharification test was performed every 10 min after the temperature reached 65 °C.

### 2.5. Filtration Speed

To assess the filtration, the cooled mash (20 °C) was brought into a funnel with a 597 ½ filter paper with 4–7 µm pore size (Whatman, Maidstone, UK), and the filtrate (wort) mass was weighed after 5, 10, 20, 40, 60, and 90 min. Only the filtrate mass after 60 min was used for the analysis.

### 2.6. Wort Extracted content, pH, Beta-Glucan Content, and Soluble Nitrogen

The extracted content and the pH of the wort samples were determined with a DMA 4500 Densimeter and Alcolyzer Plus (Anton Paar, Graz, Austria). The content of mixed linkage [(1–3)(1–4)]-β-D-glucan content was determined using the McCleary method (Megazymes, Ireland), as described before [6]. The soluble nitrogen content of the wort samples was determined via the Dumas principle [16] using a Primacs C/N Analyser (Skalar, The Netherlands) according to the ISO 16634-1:2008 method.

### 2.7. Wort Color, Free Amino Nitrogen, Polyphenol Content, and Viscosity

The color (EBC 8.5), free amino nitrogen (FAN; EBC 8.10), polyphenol content (EBC 8.12), and viscosity (EBC 8.4) of the wort samples were determined according to their appropriate EBC methods.

### 2.8. Wort Sugars

Wort sugars (glucose, fructose, sucrose, maltose, maltotriose, maltotetraose, and maltopentaose) were analyzed using high-performance anion exchange chromatography coupled with a pulsed amperometric detector (HPAEC-PAD) using a Dionex ICS-3000 system (Thermo Fisher Scientific, Sunnyvale, CA, USA), as described before [7]. Briefly, 35 μL of the sample was added to 965 μL of a deproteinization solution (25% acetonitrile) containing rhamnose as the internal standard. After centrifugation (10 min at 16,000 rpm at 4 °C), 35 µL of the supernatant was added to 965 µL of MQ water. Then, 10 μL of this dilution was injected and separated in a PA100 guard column (50 mm × 4.6 mm) followed by a PA100 analytical column (250 mm × 4.6 mm) at 30 °C. The eluent consisted of a gradient of 84 mM NaOH in ultrapure water (eluent A) and 84 mM NaOH with 250 mM sodium acetate (eluent B). An external standard curve was used for quantification.

### 2.9. Statistics

All experiments were performed in triplicate, and results were presented as the mean ± standard deviation. An ANOVA was performed to test for differences between the mashing process and wort characteristics (except for the saccharification time (min)) for barley malt and the 12 different unmalted adjuncts (10 unmalted alternative adjuncts and unmalted barley and wheat), followed by a series of post hoc pairwise comparisons using Fisher’s least significant difference (LSD) tests. These tests were performed in R 4.1.2 with a significance level of 0.05. Bivariate Spearman correlation coefficients (SCC) were calculated between the characteristics of the unmalted adjuncts as published before [6] and the resulting mashing process and wort characteristics using two-tailed significance testing at a significance level of 0.05 with SPSS software version 28.

## 3. Results

### 3.1. Congress Mashing

**Figure 1:**
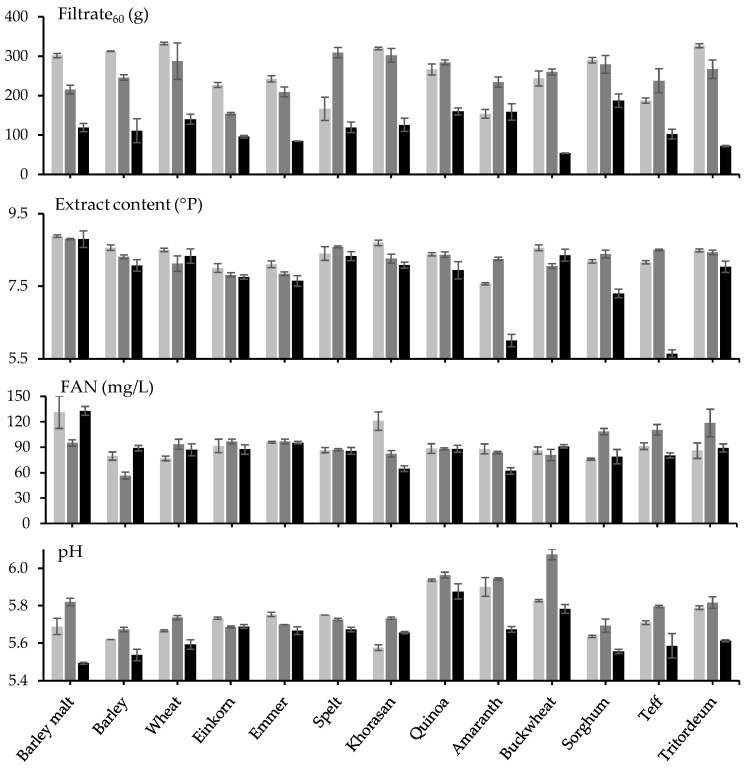
The filtrate mass after 60 min of filtration, and the density, the free amino nitrogen (FAN), and the pH of the filtrate obtained from 60% barley malt and 40% barley malt or unmalted adjunct after Congress mash (■), Congress mash with pre-gelatinization of 40% of barley malt or unmalted adjunct (■), and Evans mash (■).

Filtration of wort with 40% unmalted barley, wheat, khorasan, and tritordeum was comparable to wort with 100% barley malt, while wort with 40% unmalted einkorn, emmer, quinoa, buckwheat, and sorghum filtered slower, and wort with 40% unmalted spelt, amaranth, and teff filtered the slowest (Figure 1; Table 1). The filtrate mass after 60 min was not correlated with the starch (SCC = 0.047; *p* = 0.787) or protein (SCC = −0.153; *p* = 0.374) content of the adjunct (Table S1). The filtrate mass after 60 min was also not correlated with wort viscosity (SCC = 0.072; *p* = 0.678), which was highest with 40% unmalted buckwheat but was positively correlated with the beta-glucan content of the adjunct (SCC = 0.498; *p* = 0.002) and with the beta-glucan content in the wort (SCC = 0.390; *p* = 0.019). The latter two parameters were highly positively correlated (SCC = 0.831; *p* < 0.001).

The filtrate mass after 60 min was also positively correlated with the extracted content (SCC = 0.626; *p* < 0.001), and the maltose (SCC = 0.500; *p* = 0.002) and maltotriose (SCC = 0.358; *p* = 0.032) concentrations (albeit not with the glucose (SCC = −0.249; *p* = 0.143), maltotetraose (SCC = 0.142; *p* = 0.409), and maltopentaose concentrations (SCC = 0.006; *p* = 0.974)), but not with the FAN (SCC = −0.263; *p* = 0.122) or wort soluble protein (SCC = 0.181; *p* = 0.290). The wort soluble protein was between 3 and 5 mg/L for barley malt and all unmalted adjuncts, with the highest values found for barley malt and spelt and the lowest values found for sorghum and teff (Table 1).

The extracted content was highest for wort with 100% barley malt, followed by wort with 40% unmalted barley, wheat, spelt, khorasan, quinoa, buckwheat, and tritordeum, and the extracted content was lowest for wort with 40% unmalted einkorn, emmer, amaranth, sorghum, and teff (Figure 1; Table 1). The extracted content was positively correlated with the thousand kernel weight (TKW; SCC = 0.634; *p* < 0.001) and with the diastatic power of the adjunct (SCC = 0.445; *p* = 0.002) and negatively correlated with the gelatinization temperature (SCC = −0.425; *p* = 0.010). The extracted content was not correlated with the starch (SCC = 0.302; *p* = 0.074) or protein (SCC = 0.066; *p* = 0.704) content of the adjunct. The extracted content was also positively correlated with wort viscosity (SCC = 0.414; *p* = 0.012). The wort viscosity itself correlated positively with the soluble protein content of the wort (SCC = 0.370; *p* = 0.026) but not with the FAN (SCC = −0.247; *p* = 0.146), beta-glucan (SCC = 0.003; *p* = 0.987), glucose (SCC = −0.161; *p* = 0.348), maltose (SCC = 0.116; *p* = 0.499), maltotriose (SCC = −0.063; *p* = 0.716), maltotetraose (SCC = −0.205; *p* = 0.230), and maltopentaose (SCC = 0.154; *p* = 0.371) content of the wort.

**Figure 2:**
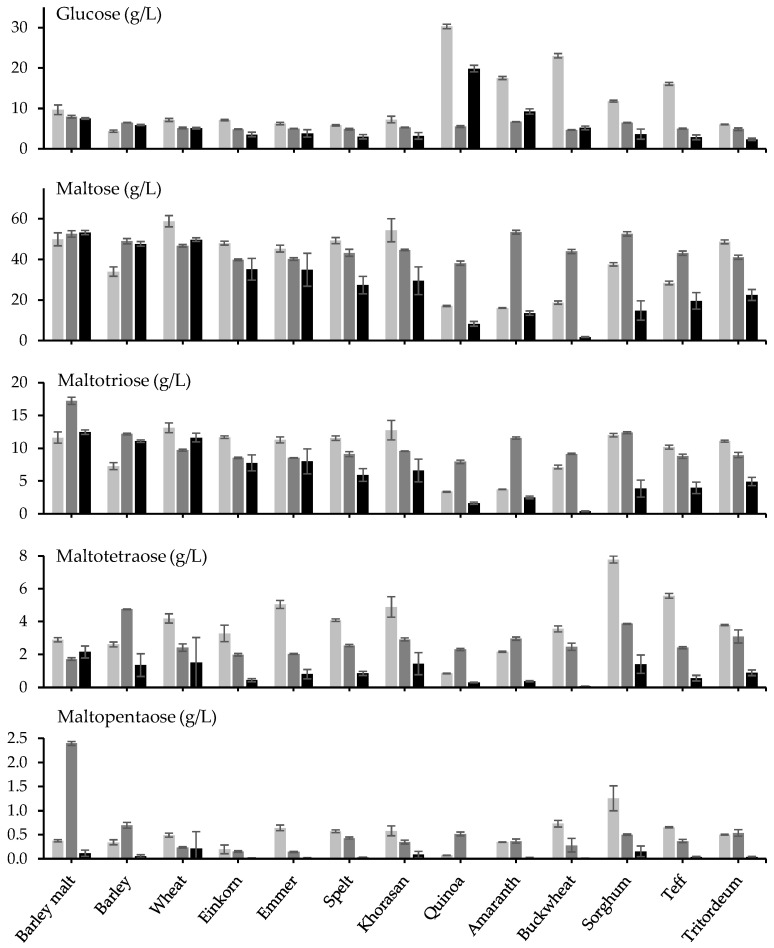
The concentrations of different sugars in wort obtained from 60% barley malt and 40% barley malt or unmalted adjunct after Congress mash (■), Congress mash with pre-gelatinization of the 40% barley malt or unmalted adjunct (■), and Evans mash (■) procedure.

The wort glucose concentration was 5–10 g/L, except for quinoa, amaranth, buckwheat, sorghum, and teff, which contained substantially higher concentrations (Figure 2; Table 1). Maltose was present at around 40–60 g/L, except for unmalted barley, quinoa, amaranth, buckwheat, sorghum, and teff, which contained substantially less maltose. Especially low concentrations of maltotriose and maltotetraose were found for quinoa and amaranth, and especially low concentrations of maltopentaose were found for einkorn and quinoa. On the other hand, especially high concentrations of maltotetraose and maltopentaose were found for sorghum. The concentrations of glucose were negatively correlated with the diastatic power (SCC = −0.766; *p* < 0.001), and more specifically, beta-amylase (SCC = −0.789; *p* < 0.001) but not alfa-amylase (SCC = −0.099; *p* = 0.565) of the adjunct. In contrast, the maltose concentrations were positively correlated with the diastatic power (SCC = 0.596; *p* < 0.001), the alfa-amylase (SCC = 0.342; *p* = 0.041), and the beta-amylase (SCC = 0.708; *p* < 0.001) content of the adjunct. The concentration of glucose correlated negatively with the concentration of maltose (SCC = −0.593; *p* < 0.001).

The wort soluble protein was positively correlated with the TKW (SCC = 0.666; *p* < 0.001), the protein content (SCC = 0.635; *p* < 0.001), and the diastatic power (SCC = 0.642; *p* < 0.001) of the adjunct (Table S1). The wort FAN content was highest for barley malt and khorasan at around 130 mg/L and was around 80 mg/L for all other adjuncts (Figure 1; Table 1). The wort FAN was also positively correlated with the protein content of the adjunct (SCC = 0.369; *p* = 0.027) but not with the soluble protein in the wort (SCC = 0.322; *p* = 0.056).

An acetate buffer was added to minimize the effect of pH, and for barley malt and most unmalted adjuncts, the pH of the wort was around 5.70 (Figure 1; Table 1). However, the pH was higher for quinoa, amaranth, and buckwheat at around 5.90; and lower for khorasan and sorghum at around 5.60. The color was low for most adjuncts at around 4 EBC but substantially higher for einkorn and teff. The color was positively correlated with polyphenols (SCC = 0.353; *p* = 0.035) and negatively correlated with adjunct protein content (SCC = −0.574; *p* < 0.001) and the wort soluble protein (SCC = −0.566; *p* < 0.001), but not with wort FAN content (SCC = −0.012; *p* = 0.942). Wort polyphenol content was highest for quinoa, barley, and barley malt and lowest for khorasan and amaranth.

Interestingly, the adjunct Ca^2+^ content was negatively correlated with the wort viscosity (SCC = −0.530; *p* < 0.001), wort soluble protein (SCC = −0.385; *p* = 0.020), filtrate after 60 min (SCC = −0.443; *p* = 0.007), and extracted content (SCC = −0.731; *p* < 0.001) (Table S1). Ca^2+^ content of the adjunct was negatively correlated with maltose (SCC = −0.432; *p* = 0.009), maltotriose (SCC = −0.389; *p* = 0.019), maltotetraose (SCC = −0.289; *p* = 0.087), and maltopentaose (SCC = −0.524; *p* = 0.001). However, the adjunct Ca^2+^ content was also negatively correlated with the adjunct starch content (SCC = −0.469; *p* = 0.004) and the TKW (SCC = −0.598; *p* < 0.001).

### 3.2. Pre-Gelatinization of the Adjunct

Pre-gelatinization generally reduced the filtrate mass after 60 min, except for spelt, quinoa, amaranth, buckwheat, and teff (Figure 1; Table 1). The filtrate mass after 60 min was again positively correlated with the extracted content (SCC = 0.549; *p* < 0.001), but no correlation with the beta-glucan content of the adjunct could be found (SCC = 0.092; *p* = 0.593) and neither with the wort FAN content (SCC = −0.126; *p* = 0.464) (Table S2).

The extracted content was generally lower after pre-gelatinization, except for spelt, quinoa, amaranth, sorghum, and teff (Figure 1; Table 1). The extracted content was not correlated anymore with the TKW (SCC = −0.058; *p* = 0.737) nor with the diastatic power (SCC = 0.173; *p* = 0.313) or starch gelatinization temperature (SCC = 0.131; *p* = 0.448) of the adjunct. After pre-gelatinization of the adjunct, the extracted content was positively correlated with the starch (SCC = 0.472; *p* = 0.004) but remained uncorrelated with the protein (SCC = −0.098; *p* = 0.568) content of the adjunct.

After pre-gelatinization, the glucose concentrations for quinoa, amaranth, buckwheat, sorghum, and teff decreased until around 5–10 g/L, whereby the maltose concentrations for these adjuncts increased until around 40–60 g/L (Figure 2; Table 1). Pre-gelatinization also increased the maltotriose concentrations for barley malt and unmalted barley, quinoa, amaranth, buckwheat, and sorghum. Overall, maltotetraose concentrations decreased after pre-gelatinization, and maltopentaose concentrations became especially high after pre-gelatinization of barley malt. After pre-gelatinization of the adjunct, the diastatic power of the adjunct was not correlated anymore with the concentrations of glucose (SCC = −0.041; *p* = 0.811) or maltose (SCC = −0.130; *p* = 0.450) in the wort (Table S2). The concentrations of glucose and maltose became positively correlated (SCC = 0.602; *p* < 0.001) after pre-gelatinization.

The wort FAN content was lower after the pre-gelatinization of barley malt, barley, and khorasan, while it became higher after the pre-gelatinization of wheat, sorghum, teff, and tritordeum (Figure 1; Table 1). The wort FAN was not correlated with the adjunct protein content (SCC = −0.226; *p* = 0.185) (Table S2). Generally, the wort pH was higher after pre-gelatinization than without pre-gelatinization of the adjunct, except for einkorn, emmer, and spelt. The adjunct Ca^2+^ content was again negatively correlated with the filtrate after 60 min (SCC = −0.626; *p* < 0.001) but not with the extracted content (SCC = −0.103; *p* = 0.550).

### 3.3. Evans Mash

The filtrate after 60 min was always lower after Evans mash than after Congress mash, except for amaranth, where both were similar (Figure 1; Table 1). In contrast to Congress mash with or without pre-gelatinization, the filtrate mass after 60 min was not correlated with the extracted content (SCC = −0.226; *p* = 0.184) nor with the beta-glucan content of the adjunct (SCC = −0.283; *p* = 0.094) but was correlated negatively with the FAN (SCC = −0.455; *p* = 0.005) (Table S3).

The extracted content was generally lower after Evans mash than after Congress mash, especially for amaranth and teff (Figure 1; Table 1). The extracted content was positively correlated with the TKW of the adjunct (SCC = 0.667; *p* < 0.001) and FAN of the wort (SCC = 0.345; *p* = 0.040) but not with the starch (SCC = 0.159; *p* = 0.356) or protein (SCC = 0.271; *p* = 0.110) content of the adjunct. The extracted content was also positively correlated with the diastatic power (SCC = 0.439; *p* = 0.007) of the adjunct but not with the alpha-amylase (SCC = 0.254; *p* = 0.135) or beta-amylase (SCC = 0.273; *p* = 0.108) content separately (Table S3). Furthermore, the extracted content was negatively correlated with the starch gelatinization temperature of the adjunct (SCC = −0.497; *p* = 0.002).

The concentrations of glucose were generally lower after Evans mashing than after Congress mashing, and this effect was especially pronounced for quinoa, amaranth, buckwheat, and sorghum (Figure 2; Table 1). For barley malt and unmalted barley, Evans mashing resulted in slightly higher maltose and maltotriose concentrations, but for all other adjuncts, the concentrations of maltose and maltotriose were lower after Evans mashing. Maltotetraose and maltopentaose concentrations were always lower after Evans mashing than after Congress mashing. The glucose concentrations were not correlated with the diastatic power (SCC = −0.196; *p* = 0.252) or the alpha-amylase (SCC = −0.101; *p* = 0.558) content of the adjuncts but were negatively correlated with their beta-amylase content (SCC = −0.420; *p* = 0.011) (Table S3). In contrast, the maltose concentrations correlated positively with the diastatic power (SCC = 0.591; *p* < 0.001) and the beta-amylase (SCC = 0.693; *p* < 0.001), but not the alpha-amylase (SCC = 0.267; *p* = 0.116) content of the adjunct. In contrast to the Congress mash with or without pre-gelatinization, the concentrations of glucose and maltose were not correlated after Evans mashing (SCC = −0.082; *p* = 0.633).

The wort FAN content after Evans mash was similar to after Congress mash, except for khorasan, where the wort contained less FAN after Evans mashing (Figure 1; Table 1). The wort FAN content was not correlated with the adjunct protein content (SCC = −0.282; *p* = 0.096) (Table S3). The pH of the wort after Evans mashing was generally lower than the pH after Congress mashing, except for khorasan. The Ca^2+^ content of the adjunct was not correlated with the filtrate after 60 min (SCC = 0.139; *p* = 0.419) but was negatively correlated with the extracted content (SCC = −0.679; *p* < 0.001).

## 4. Discussion

The experiments performed for this study provide insight into the behavior of einkorn, emmer, spelt, khorasan, quinoa, amaranth, buckwheat, sorghum, teff, and tritordeum during wort production on a laboratory scale. Combining this dataset with previously published results about the physical, physicochemical, and chemical properties of these adjuncts allowed a deeper understanding of their impact on the mashing process and the resulting wort characteristics [6].

With a Congress mashing procedure, the extracted content with 40% unmalted adjuncts was always lower than the extracted content resulting from 100% barley malt. This indicated incomplete starch breakdown when unmalted alternative adjuncts were used, as barley malt contained only 55% (dm) starch while most adjuncts contained >55% starch (except for einkorn and emmer, which contained an unusually high amount of husk) [6]. Incomplete starch breakdown during mashing with these adjuncts was further confirmed by the lack of correlation between the adjunct starch content and the extracted content of the resulting wort. This may either be caused by incomplete starch gelatinization, as the extracted content correlated negatively with the onset starch gelatinization temperature of the adjunct, or by insufficient enzyme activity, as the extracted content was positively correlated with the diastatic power of the adjunct.

The onset starch gelatinization temperature was indeed higher for all unmalted alternative adjuncts than for unmalted barley or wheat. When pre-gelatinization of the unmalted adjuncts was performed to eliminate the effect of different onset starch gelatinization temperatures, the extracted content generally decreased, except for spelt, amaranth, sorghum, and teff. The latter three were indeed characterized by the highest starch onset gelatinization temperatures (T_gel_ > 65 °C) and would thus benefit most from a pre-gelatinization step. In contrast, spelt was characterized by a modest starch onset gelatinization temperature of 60.8 °C and would theoretically not benefit from a pre-gelatinization step. Care has to be taken when using starch onset gelatinization temperatures, as it was shown that small starch granules have higher onset gelatinization temperatures, which implies that the size distribution of the starch granules influences the starch gelatinization process [17,18]. Nevertheless, as reported previously, the starch onset gelatinization temperatures were in line with the results of the Congress mash with or without pre-gelatinization in this study and thus provided valuable information. Additionally, adjuncts with T_gel_ > 65 °C (amaranth, sorghum, and teff) resulted in especially low extracted contents with the Evans mashing procedure (isothermic mashing at 65 °C), further confirming the value of the onset starch gelatinization temperature. Pre-gelatinization promoted starch gelatinization but also inactivated endogenous enzymes. The higher diastatic power of the unmalted alternative adjunct increased the wort extracted content for the Congress mash and the Evans mash but not the extracted content for the Congress mash with pre-gelatinization. This adverse effect will probably be less pronounced for adjuncts with low diastatic power, and the effect of pre-gelatinization was indeed most beneficial for those unmalted adjuncts with the lowest diastatic power.

Insufficient starch-degrading enzymes could also have caused incomplete starch breakdown, as the extracted content correlated positively with the diastatic power of the adjunct (although not with the individual alpha- or beta-amylase contents). Alpha-amylase will probably contribute most to the starch solubilization, but the amount of this enzyme was negligible in the unmalted adjuncts. In contrast, the amount of beta-amylase in these unmalted adjuncts was substantial and differed significantly between the different adjuncts. However, diastatic power or alpha- and beta-amylase measurements may not encompass all enzymatic activity involved in starch breakdown. More specifically, proteolytic enzymes (but also cytolytic enzymes, albeit to a lesser extent) were proven to enhance starch breakdown during mashing, both via improved starch gelatinization as well as via the degradation of inhibitors of beta-amylase and limit-dextranase [19].

The sugar composition of the wort varied widely between Congress mash with different unmalted alternative adjuncts. The concentration of maltose in the wort increased with the beta-amylase content of the unmalted adjunct, both after Congress mash and after Evans mash, but not after Congress mash with pre-gelatinization, indicating the inactivation of endogenous enzymes during pre-gelatinization. Mashing with quinoa, amaranth, buckwheat, sorghum, and teff resulted in unusually high concentrations of glucose combined with unusually low concentrations of maltose and maltotriose. After pre-gelatinization, the glucose concentrations decreased, and the maltose and maltotriose concentrations increased, indicating the presence of substantial alpha-glucosidase activity in these unmalted alternative adjuncts, which were inactivated upon pre-gelatinization. The presence of these enzymes in these adjuncts has indeed been reported in the past [20,21,22,23,24], except for teff. There may be different forms of alpha-glucosidases, as previously reported for millet [25], where the alpha-glucosidases had optimal activity at pH 3.5 to 6.0 and a temperature of 60 °C. Buckwheat alpha-glucosidases preferred shorter maltodextrins over longer maltodextrins (DP = 13) [24], whereas millet alpha-glucosidases had higher affinity for polysaccharides than for maltose [25].

The filtration was not influenced by wort viscosity but proceeded faster when the extracted content of the wort was higher, indicating that more extensive macromolecule breakdown resulted in better filtration. This was probably mainly attributable to carbohydrate breakdown, not protein breakdown, as the filtration was faster with higher maltose and maltotriose concentrations in the wort but was not significantly influenced by the FAN or the soluble protein. The association between the higher extracted content and faster filtration was not present for the Evans mash, probably because (part of) the starch was only gelatinized during mashing-out at 74 °C. This starch could not contribute any more to the extracted content and might have hampered filtration. Furthermore, filtration proceeded slower when the concentration of beta-glucan in the wort was higher, as was expected [26]. However, only unmalted barley contained substantial amounts of beta-glucan [6], also reflected in the wort composition. The viscosity did not influence the filtration but might be of interest because it may influence the mouthfeel of beer [27], especially as it is one of the main shortcomings of non- and low-alcoholic beers (NABLAB) [28]. In this study, the viscosity was higher when a more soluble protein was present in the wort. Previous research suggested that soluble proteins of 2–20 kDa can indeed contribute to beer’s mouthfeel by increasing softness and smoothness and decreasing astringency [29]. In this study, wort with more soluble protein was obtained with unmalted adjuncts with higher TKW, protein content, and diastatic power.

Ca^2+^ is a cofactor of amylases, promotes hot break formation during wort boiling, promotes flocculation after fermentation, prevents haze formation during beer storage, and promotes the removal of oxalates during the brewing process (which may form beer stone) [30,31,32]. Adding extra Ca^2+^ at the start of the mashing process is thus common practice in the brewing industry, which is also incorporated by the Evans mash procedure [15]. The positive impact of Ca^2+^ on the thermostability of barley malt amylase enzymes has been known for a long time [33], but there are also indications that excessive Ca^2+^ ions might decrease amylase activity in barley malt [34] and in rice malt [35]. In this study, unmalted adjuncts with higher Ca^2+^ concentrations resulted in lower extracted contents (and in particular sugars such as maltose and maltotriose) and slower filtration, together with lower wort soluble protein and lower viscosity. More Ca^2+^ seemed thus associated with less macromolecule (carbohydrate and protein) degradation. The adjuncts with the highest Ca^2+^ concentration were amaranth, teff, einkorn, and emmer, and those with the lowest Ca^2+^ concentration were buckwheat, khorasan, spelt, and wheat [6]. For the Evans mash, whereby extra Ca^2+^ was added during the mashing process, the effect of the Ca^2+^ content in the adjunct on the extracted content of the wort became less pronounced. Ca^2+^ is known to promote the formation of macromolecule complexes, similar to the role of Ca^2+^ in membrane fouling [36]. These complexes might have precipitated in the filter bed, which might have hampered their degradation, reduced wort viscosity, and hampered filtration. There are indeed indications that Ca^2+^ reduces the dissolution of protein from malt into the wort [37]. Nevertheless, it has to be noted that the Ca^2+^ concentration was lower when the TKW was higher, which might also have influenced the results, as unmalted adjuncts with higher TKW resulted in a better mashing process (faster saccharification time, faster filtration, and higher extracted content).

## 5. Conclusions

In conclusion, wort production was possible with all 10 unmalted alternative cereals and pseudocereals. However, macromolecule breakdown was not complete for many unmalted alternative cereals and pseudocereals. Overall, Congress mash yielded the highest extracts and fastest filtration, followed by the Congress mash with pre-gelatinization, whereas Evans mash resulted in the lowest extracted content and filtration. The alternative adjuncts generally needed higher mashing temperatures than barley malt to obtain satisfactory extract and filtration, especially when the onset starch gelatinization temperature was >65 °C. In this case, a pre-gelatinization step could improve the extracted content and the filtration, whereas, for most adjuncts, a pre-gelatinization was not advantageous for extracted content or the filtration. This could be explained by the inactivation of endogenous enzymes during the pre-gelatinization step, which contributed substantially to the mashing process. Furthermore, these endogenous enzymes have a substantial impact on the resulting wort characteristics, especially for quinoa, amaranth, buckwheat, sorghum, or teff, whereby a wort especially rich in glucose and poor in maltose and maltotriose was obtained, probably because of the presence of endogenous alpha-glucosidases. Pre-gelatinization inactivated these enzymes, which resulted in a decrease in the glucose concentrations and a concomitant increase in the maltose and maltotriose concentrations. Most alternative unmalted adjuncts resulted in wort with lower FAN (and lower wort soluble protein) despite their higher protein content than barley malt. High wort soluble protein and wort beta-glucan were associated with high wort viscosity. However, wort with 40% unmalted buckwheat had especially high viscosity despite low beta-glucan content and moderate soluble protein in the wort. These results create novel possibilities to meet the increasing consumer demand for gluten-free and specialty beers and may contribute to the rational selection of novel and more sustainable crops for the brewing industry.

## Figures and Tables

**Table 1:** Characteristics of the mashing process and the wort produced from 60% barley malt and 40% barley malt or unmalted adjunct according to a Congress mash, a Congress mash with pre-gelatinization of the 40% barley malt or unmalted adjunct, and an Evans mash. The data are presented as the mean ± the standard deviation. Significant differences (*p* < 0.05) were calculated for all parameters and are indicated with different superscript letters (a, b, c, d, e, f, g, h, i, j, and k).

		Barley Malt	Barley	Wheat	Einkorn	Emmer	Spelt	Khorasan	Quinoa	Amaranth	Buckwheat	Sorghum	Teff	Tritordeum
**Congress mash**	**Saccharification time (min)**	20 ^c^	20 ^c^	20 ^c^	15 ^d^	15 ^d^	20 ^c^	15 ^d^	30 ^b^	15 ^d^	20 ^c^	60 ^a^	60 ^a^	20 ^c^
	**Filtrate mass after 60 min (g)**	302 ± 6 ^bc^	313 ± 1 ^ab^	332 ± ^3 a^	227 ± 6 ^e^	242 ± 8 ^e^	166 ± 30 ^g^	320 ± 3 ^ab^	266 ± 14 ^d^	154 ± 11 ^g^	243 ± 19 ^e^	290 ± 7 ^c^	187 ± 7 ^f^	327 ± 5 ^a^
	**Extracted content (°P)**	8.88 ± 0.03 ^a^	8.56 ± 0.07 ^bc^	8.50 ± 0.05 ^cd^	8.00 ± 0.12 ^f^	8.11 ± 0.09 ^ef^	8.40 ± 0.19 ^d^	8.69 ± 0.08 ^b^	8.38 ± 0.04 ^d^	7.57 ± 0.03 ^g^	8.56 ± 0.08 ^bc^	8.18 ± 0.05 ^e^	8.16 ± 0.04 ^e^	8.49 ± 0.04 ^cd^
	**pH**	5.69 ± 0.04 ^gh^	5.62 ± 0.00 ^j^	5.67 ± 0.01 ^hi^	5.73 ± 0.01 ^ef^	5.75 ± 0.01 ^e^	5.75 ± 0.00 ^e^	5.58 ± 0.02 ^k^	5.94 ± 0.01 ^a^	5.90 ± 0.05 ^b^	5.83 ± 0.01 ^c^	5.64 ± 0.01 ^ij^	5.71 ± 0.01 ^fg^	5.79 ± 0.01 ^d^
	**Free amino nitrogen (mg/L)**	131 ± 19 ^a^	80 ± 5 ^cd^	77 ± 3 ^d^	92 ± 8 ^bc^	96 ± 1 ^b^	87 ± 3 ^bcd^	121 ± 11 ^a^	88 ± 6 ^bcd^	88 ± 6 ^bcd^	86 ± 4 ^bcd^	76 ± 1 ^d^	91 ± 4 ^bc^	86 ± 9 ^bcd^
	**Glucose (g/L)**	9.7 ± 1.2 ^f^	4.4 ± 0.3 ^j^	7.1 ± 0.4 ^g^	7.1 ± 0.2 ^gh^	6.3 ± 0.3 ^hi^	5.8 ± 0.2 ^i^	7.2 ± 0.8 ^g^	30.3 ± 0.6 ^a^	17.5 ± 0.4 ^c^	23.0 ± 0.5 ^b^	11.8 ± 0.2 ^e^	16.1 ± 0.4 ^d^	6.0 ± 0.0 ^i^
	**Maltose (g/L)**	49.9 ± 3.2 ^c^	34.0 ± 2.3 ^e^	58.8 ± 2.8 ^a^	48.0 ± 0.9 ^cd^	45.3 ± 1.7 ^d^	49.2 ± 1.5 ^c^	54.3 ± 5.7 ^b^	17.1 ± 0.2 ^g^	16.0 ± 0.2 ^g^	18.7 ± 0.8 ^g^	37.6 ± 0.8 ^e^	28.3 ± 0.9 ^f^	48.6 ± 1.0 ^cd^
	**Maltotriose (g/L)**	11.6 ± 0.9 ^c^	7.3 ± 0.5 ^e^	13.1 ± 0.7 ^a^	11.7 ± 0.2 ^c^	11.3 ± 0.5 ^c^	11.5 ± 0.4 ^c^	12.7 ± 1.5 ^ab^	3.4 ± 0.1 ^f^	3.7 ± 0.0 ^f^	7.1 ± 0.3 ^e^	12.0 ± 0.3 ^bc^	10.2 ± 0.3 ^d^	11.1 ± 0.2 ^cd^
	**Maltotetraose (g/L)**	2.89 ± 0.13 ^gh^	2.61 ± 0.15 ^h^	4.19 ± 0.28 ^d^	3.28 ± 0.50 ^fg^	5.05 ± 0.24 ^c^	4.09 ± 0.08 ^d^	4.90 ± 0.63 ^c^	0.84 ± 0.01 ^j^	2.16 ± 0.04 ^i^	3.55 ± 0.18 ^ef^	7.79 ± 0.22 ^a^	5.57 ± 0.14 ^b^	3.80 ± 0.04 ^de^
	**Maltopentaose (g/L)**	0.37 ± 0.02 ^fgh^	0.34 ± 0.05 ^h^	0.49 ± 0.04 ^efg^	0.19 ± 0.09 ^i^	0.64 ± 0.06 ^bcd^	0.57 ± 0.03 ^cde^	0.58 ± 0.10 ^bcde^	0.07 ± 0.00 ^i^	0.35 ± 0.00 ^gh^	0.73 ± 0.07 ^b^	1.25 ± 0.26 ^a^	0.65 ± 0.01 ^bc^	0.50 ± 0.01 ^def^
	**Viscosity (mPa·s)**	1.31 ± 0.04 ^d^	1.30 ± 0.03 ^de^	1.32 ± 0.03 ^cd^	1.25 ± 0.03 ^e^	1.25 ± 0.03 ^e^	1.40 ± 0.03 ^b^	1.29 ± 0.04 ^de^	1.25 ± 0.03 ^e^	1.32 ± 0.03 ^cd^	1.67 ± 0.06 ^a^	1.27 ± 0.03 ^de^	1.25 ± 0.03 ^e^	1.38 ± 0.03 ^bc^
	**Soluble protein (mg/L)**	5.2 ± 0.1 ^a^	4.2 ± 0.1 ^f^	4.8 ± 0.1 ^cd^	4.5 ± 0.1 ^e^	4.9 ± 0.1 ^bc^	5.3 ± 0.1 ^a^	5.0 ± 0.1 ^b^	4.6 ± 0.1 ^de^	4.5 ± 0.1 ^e^	4.5 ± 0.1 ^e^	2.9 ± 0.1 ^h^	3.8 ± 0.1 ^g^	4.9 ± 0.1 ^bc^
	**Beta-glucan (mg/L)**	48 ± 4 ^bc^	449 ± 57 ^a^	77 ± 4 ^b^	52 ± 13 ^bc^	72 ± 9 ^b^	75 ± 21 ^b^	46 ± 6 ^bc^	29 ± 6 ^cd^	6 ± 6 ^d^	7 ± 8 ^d^	34 ± 13 ^cd^	34 ± 15 ^cd^	77 ± 45 ^b^
	**Color (EBC)**	6.0 ± 0.9 ^c^	5.5 ± 0.2 ^c^	3.9 ± 0.1 ^efg^	8.5 ± 0.1 ^a^	4.3 ± 0.5 ^de^	3.0 ± 0.3 ^h^	3.7 ± 0.4 ^fg^	5.6 ± 0.1 ^c^	3.4 ± 0.2 ^gh^	3.7 ± 0.2 ^fg^	4.8 ± 0.2 ^d^	7.4 ± 0.3 ^b^	4.2 ± 0.1 ^def^
	**Polyphenols (mg/L)**	70.5 ± 5.0 ^c^	79.3 ± 3.7 ^b^	39.9 ± 3.7 ^fg^	44.8 ± 3.3 ^ef^	47.3 ± 4.8 ^e^	44.3 ± 3.0 ^ef^	22.4 ± 1.3 ^h^	102.2 ± 3.8 ^a^	23.8 ± 0.0 ^h^	65.3 ± 1.9 ^c^	57.4 ± 6.2 ^d^	38.8 ± 2.1 ^fg^	36.4 ± 4.1 ^g^
**Congress mash +** **pre-gelatinization**	**Saccharification time (min)**	20 ^c^	20 ^c^	15 ^d^	15 ^d^	15 ^d^	20 ^c^	15 ^d^	50 ^b^	15 ^d^	15 ^d^	60 ^a^	60 ^a^	20 ^c^
	**Filtrate mass after 60 min (g)**	215 ± 11 ^ef^	246 ± 7 ^cde^	287 ± 46 ^ab^	154 ± 3 ^g^	209 ± 13 ^f^	309 ± 13 ^a^	302 ± 17 ^a^	284 ± 6 ^ab^	234 ± 13 ^def^	260 ± 8 ^bcd^	279 ± 22 ^abc^	238 ± 30 ^def^	267 ± 23 ^bcd^
	**Extracted content (°P)**	8.80 ± 0.02 ^a^	8.31 ± 0.05 ^de^	8.12 ± 0.21 ^fg^	7.81 ± 0.06 ^h^	7.84 ± 0.05 ^h^	8.59 ± 0.02 ^b^	8.26 ± 0.12 ^ef^	8.37 ± 0.08 ^cde^	8.26 ± 0.05 ^ef^	8.06 ± 0.07 ^g^	8.39 ± 0.11 ^cde^	8.50 ± 0.02 ^bc^	8.43 ± 0.06 ^cd^
	**pH**	5.82 ± 0.02 ^c^	5.67 ± 0.01 ^f^	5.74 ± 0.01 ^d^	5.69 ± 0.01 ^f^	5.70 ± 0.00 ^ef^	5.73 ± 0.01 ^de^	5.73 ± 0.01 ^d^	5.96 ± 0.02 ^b^	5.94 ± 0.01 ^b^	6.07 ± 0.03 ^a^	5.69 ± 0.04 ^f^	5.80 ± 0.01 ^c^	5.82 ± 0.03 ^c^
	**Free amino nitrogen (mg/L)**	95 ± 4 ^c^	57 ± 4 ^f^	93 ± 6 ^cd^	97 ± 3 ^c^	97 ± 3 ^c^	87 ± 1 ^cde^	82 ± 4 ^e^	88 ± 1 ^cde^	84 ± 1 ^de^	81 ± 7 ^e^	108 ± 4 ^b^	111 ± 6 ^ab^	119 ± 16 ^a^
	**Glucose (g/L)**	8.0 ± 0.3 ^a^	6.5 ± 0.1 ^b^	5.2 ± 0.2 ^de^	4.8 ± 0.1 ^fg^	5.0 ± 0.1 ^ef^	4.9 ± 0.2 ^fg^	5.3 ± 0.1 ^cd^	5.5 ± 0.2 ^c^	6.7 ± 0.1 ^b^	4.7 ± 0.1 ^g^	6.5 ± 0.1 ^b^	5.0 ± 0.1 ^ef^	4.9 ± 0.3 ^fg^
	**Maltose (g/L)**	52.5 ± 1.6 ^a^	49.0 ± 1.3 ^b^	46.7 ± 0.6 ^c^	39.7 ± 0.4 ^ef^	40.1 ± 0.6 ^e^	43.2 ± 1.7 ^d^	44.6 ± 0.4 ^d^	38.1 ± 1.0 ^f^	53.4 ± 0.9 ^a^	43.9 ± 1.0 ^d^	52.5 ± 1.1 ^a^	43.0 ± 1.1 ^d^	41.0 ± 1.0 ^e^
	**Maltotriose (g/L)**	17.2 ± 0.6 ^a^	12.2 ± 0.1 ^b^	9.7 ± 0.2 ^d^	8.5 ± 0.1 ^g^	8.5 ± 0.0 ^g^	9.1 ± 0.4 ^f^	9.6 ± 0.0 ^de^	7.9 ± 0.3 ^h^	11.6 ± 0.2 ^c^	9.2 ± 0.1 ^ef^	12.4 ± 0.1 ^b^	8.8 ± 0.3 ^fg^	9.0 ± 0.4 ^fg^
	**Maltotetraose (g/L)**	1.72 ± 0.08 ^f^	4.75 ± 0.02 ^a^	2.42 ± 0.22 ^d^	1.98 ± 0.08 ^e^	2.03 ± 0.03 ^e^	2.54 ± 0.07 ^d^	2.92 ± 0.09 ^c^	2.30 ± 0.07 ^d^	2.96 ± 0.10 ^c^	2.46 ± 0.22 ^d^	3.86 ± 0.03 ^b^	2.41 ± 0.06 ^d^	3.10 ± 0.40 ^c^
	**Maltopentaose (g/L)**	2.39 ± 0.04 ^a^	0.70 ± 0.06 ^b^	0.24 ± 0.01 ^gh^	0.15 ± 0.02 ^hi^	0.14 ± 0.01 ^i^	0.43 ± 0.02 ^de^	0.35 ± 0.04 ^ef^	0.51 ± 0.04 ^cd^	0.37 ± 0.04 ^ef^	0.28 ± 0.14 ^fg^	0.50 ± 0.02 ^cd^	0.37 ± 0.03 ^ef^	0.54 ± 0.07 ^c^
**Evans mash**	**Saccharification time (min)**	5 ^c^	10 ^b^	10 ^b^	5 ^c^	5 ^c^	10 ^b^	10 ^b^	30 ^a^	10 ^b^	10 ^b^	10 ^b^	5 ^c^	5 ^c^
	**Filtrate mass after 60 min (g)**	119 ± 11 ^cde^	111 ± 31 ^de^	140 ± 13 ^bc^	96 ± 3 ^efg^	84 ± 1 ^fg^	119 ± 14 ^cde^	126 ± 17 ^cd^	160 ± 9 ^b^	158 ± 21 ^b^	54 ± 1 ^h^	187 ± 17 ^a^	102 ± 13 ^def^	72 ± 2 ^gh^
	**Extracted content (°P)**	8.80 ± 0.23 ^a^	8.07 ± 0.16 ^cd^	8.33 ± 0.20 ^bc^	7.75 ± 0.06 ^ef^	7.65 ± 0.14 ^f^	8.33 ± 0.13 ^bc^	8.08 ± 0.09 ^cd^	7.94 ± 0.24 ^de^	6.00 ± 0.17 ^h^	8.36 ± 0.17 ^b^	7.30 ± 0.12 ^g^	5.64 ± 0.10 ^i^	8.03 ± 0.16 ^d^
	**pH**	5.49 ± 0.01 ^h^	5.54 ± 0.03 ^gh^	5.59 ± 0.03 ^ef^	5.69 ± 0.01 ^c^	5.67 ± 0.02 ^c^	5.67 ± 0.01 ^c^	5.66 ± 0.01 ^cd^	5.88 ± 0.04 ^a^	5.67 ± 0.02 ^c^	5.78 ± 0.02 ^b^	5.56 ± 0.01 ^fg^	5.59 ± 0.07 ^ef^	5.61 ± 0.01 ^de^
	**Free amino nitrogen (mg/L)**	133 ± 5 ^a^	89 ± 3 ^bc^	87 ± 7 ^cd^	87 ± 5 ^cd^	96 ± 1 ^b^	86 ± 4 ^cde^	65 ± 3 ^f^	88 ± 4 ^bcd^	62 ± 4 ^f^	91 ± 2 ^bc^	79 ± 8 ^e^	80 ± 3 ^de^	89 ± 5 ^bc^
	**Glucose (g/L)**	7.5 ± 0.2 ^c^	5.9 ± 0.1 ^d^	5.1 ± 0.2 ^d^	3.6 ± 0.6 ^e^	3.8 ± 0.9 ^e^	3.0 ± 0.5 ^ef^	3.2 ± 0.8 ^ef^	19.9 ± 0.8 ^a^	9.2 ± 0.^6 b^	5.2 ± 0.5 ^d^	3.6 ± 1.2 ^e^	2.8 ± 0.6 ^ef^	2.3 ± 0.3 ^f^
	**Maltose (g/L)**	53.2 ± 1.0 ^a^	47.6 ± 1.1 ^a^	49.7 ± 0.9 ^a^	35.1 ± 5.3 ^b^	34.9 ± 8.1 ^b^	27.3 ± 4.3 ^cd^	29.5 ± 6.8 ^bc^	8.2 ± 1.1 ^gh^	13.5 ± 1.1 ^fg^	1.7 ± 0.2 ^h^	14.8 ± 4.8 ^fg^	19.6 ± 4.0 ^ef^	22.4 ± 2.7 ^de^
	**Maltotriose (g/L)**	12.4 ± 0.3 ^a^	11.1 ± 0.2 ^a^	11.6 ± 0.7 ^a^	7.8 ± 1.2 ^b^	8.0 ± 1.9 ^b^	5.9 ± 1.0 ^cd^	6.6 ± 1.7 ^bc^	1.6 ± 0.2 ^gh^	2.5 ± 0.2 ^fg^	0.4 ± 0.1 ^h^	3.9 ± 1.3 ^ef^	4.0 ± 0.9 ^ef^	4.9 ± 0.6 ^de^
	**Maltotetraose (g/L)**	2.14 ± 0.36 ^a^	1.35 ± 0.69 ^abcd^	1.50 ± 1.53 ^ab^	0.44 ± 0.09 ^de^	0.80 ± 0.27 ^bcde^	0.84 ± 0.12 ^bcde^	1.43 ± 0.67 ^abc^	0.30 ± 0.02 ^e^	0.38 ± 0.04 ^e^	0.06 ± 0.02 ^e^	1.40 ± 0.56 ^abc^	0.56 ± 0.17 ^cde^	0.88 ± 0.17 ^bcde^
	**Maltopentaose (g/L)**	0.11 ± 0.06	0.05 ± 0.03	0.22 ± 0.35	0.01 ± 0.00	0.02 ± 0.00	0.03 ± 0.01	0.09 ± 0.06	0.01 ± 0.00	0.02 ± 0.00	0.01 ± 0.00	0.16 ± 0.11	0.03 ± 0.02	0.04 ± 0.01

## Data Availability

The data used to support the findings of this study can be made available by the corresponding author upon request.

## Supplementary Materials

The following supporting information can be downloaded at: https://www.mdpi.com/article/10.3390/foods12234206/s1, Table S1: Bivariate Spearman correlation coefficients with their corresponding two-tailed significances be-tween characteristics of 12 unmalted adjuncts (barley, wheat, einkorn, emmer, spelt, khorasan, quinoa, ama-ranth, buckwheat, sorghum, teff and tritordeum) [thousand kernel weight (TKW, g), starch (% dm), protein (% dm), fat (% dm), beta-glucan (% dm), magnesium (Mg, mg/kg dm), potassium (K, mg/kg dm), calcium (Ca, mg/kg dm), phosphorus (P, mg/kg dm), diastatic power (°WK), alpha-amylase (CU/g dm), beta-amylase (B3U/g dm) and gelatinization temperature (°C)], the Congress mashing process characteristics with 60% barley malt and 40% unmalted adjunct [saccharification time (min) and filtrate mass after 60 min (g)] and the resulting wort charac-teristics [extracted content (°P), pH, free amino nitrogen (FAN, mg/L), glucose (g/L), maltose (g/L), maltotriose (g/L), maltotetraose (g/L), maltopentaose (g/L), wort viscosity (mPa·s), polyphenol content (mg/L), soluble protein (g/L), wort beta-glucan (mg/L) and wort color (EBC)]; Table S2: Bivariate Spearman correlation coefficients with their corresponding two-tailed significances between characteristics of 12 unmalted adjuncts (barley, wheat, einkorn, emmer, spelt, khorasan, quinoa, amaranth, buckwheat, sorghum, teff and tritordeum) [thousand kernel weight (TKW, g), starch (% dm), protein (% dm), fat (% dm), beta-glucan (% dm), magnesium (Mg, mg/kg dm), potassium (K, mg/kg dm), calcium (Ca, mg/kg dm), phosphorus (P, mg/kg dm), diastatic power (°WK), alpha-amylase (CU/g dm), beta-amylase (B3U/g dm) and gelatinization temperature (°C)], the Congress mashing process characteristics with 60% barley malt and 40% pregelatinized unmalted adjunct [saccharification time (min) and filtrate mass after 60 min (g)] and the resulting wort characteristics [extracted content (°P), pH, free amino nitrogen (FAN, mg/L), glucose (g/L), maltose (g/L), maltotriose (g/L), maltotetraose (g/L) and maltopentaose (g/L)]; Table S3: Bivariate Spearman correlation coefficients with their corresponding two-tailed significances between characteristics of 12 unmalted adjuncts (barley, wheat, einkorn, emmer, spelt, khorasan, quinoa, amaranth, buckwheat, sorghum, teff and tritordeum) [thousand kernel weight (TKW, g), starch (% dm), protein (% dm), fat (% dm), beta-glucan (% dm), magnesium (Mg, mg/kg dm), potassium (K, mg/kg dm), calcium (Ca, mg/kg dm), phosphorus (P, mg/kg dm), diastatic power (°WK), alpha-amylase (CU/g dm), beta-amylase (B3U/g dm) and gelatinization temperature (°C)], the Evans mashing process characteristics with 60% barley malt and 40% unmalted adjunct [saccharification time (min) and filtrate mass after 60 min (g)] and the resulting wort characteristics [extracted content (°P), pH, free amino nitrogen (FAN, mg/L), glucose (g/L), maltose (g/L), maltotriose (g/L), maltotetraose (g/L), and maltopentaose (g/L)].
