# Supplementary material for: The Impact of 10 Unmalted Alternative Adjuncts on Wort Characteristics
Source: Foods. 2023 Nov 22;12(23):4206. doi: 10.3390/foods12234206 (PMC10706799; doi:10.3390/foods12234206)
Supplement: Supplementary file 1 [file foods-12-04206-s001.zip › foods-2699823-supplementary.pdf]

Table S1. Bivariate Spearman correlation coefficients with their corresponding two-tailed significances between characteristics of 12 unmalted adjuncts (barley, wheat, einkorn, emmer, spelt, khorasan, quinoa, amaranth, buckwheat, sorghum, teff and tritordeum) [thousand kernel weight (TKW, g), starch (% dm), protein (% dm), fat (% dm), beta-glucan (% dm), magnesium (Mg, mg/kg dm), potassium (K, mg/kg dm), calcium (Ca, mg/kg dm), phosphorus (P, mg/kg dm), diastatic power (°WK), alpha-amylase (CU/g dm), beta-amylase (B3U/g dm) and gelatinization temperature (°C)], the Congress mashing process characteristics with 60% barley malt and 40% unmalted adjunct [saccharification time (min) and filtrate mass after 60 min (g)] and the resulting wort characteristics [extract content (°P), pH, free amino nitrogen (FAN, mg/L), glucose (g/L), maltose (g/L), maltotriose (g/L), maltotetraose (g/L), maltopentaose (g/L), wort viscosity (mPa·s), polyphenol content (mg/L), soluble protein (g/L), wort beta-glucan (mg/L) and wort color (EBC)].

	TKW (g dm)	Starch (% dm)	Protein (% dm)	Fat (% dm)	Beta-glucan (% dm)	Mg (mg/kg dm)	K (mg/kg dm)	Ca (mg/kg dm)	P (mg/kg dm)	Diastatic power (°WK)	Alpha-amylase (CU/g dm)	Beta-amylase (B3U/g dm)	Gelatinization T (°C)	C-Saccharification time (min)	C-Filtrate mass after 60 min (g)	C-Extract content (°P)	C-pH	C-FAN (mg/L)	C-Glucose (g/L)	C-Maltose (g/L)	C-Maltotriose (g/L)	C-Maltotetraose (g/L)	C-Maltopentaose (g/L)	C-Viscosity (mPa·s)	C-Polyphenol content (mg/L)	C-Soluble protein (g/L)	C-Beta-glucan (mg/L)	C-Color (EBC)
TKW (g dm)	1.000	-0.129	0.404	-0.810	0.772	-0.684	-0.077	-0.598	-0.319	0.835	0.197	0.714	-0.712	-0.377	0.513	0.634	-0.502	0.006	-0.691	0.822	0.579	0.126	-0.007	0.334	-0.187	0.666	0.639	-0.359
	.	0.453	0.015	<0.001	<0.001	<0.001	0.655	<0.001	0.058	<0.001	0.250	<0.001	<0.001	0.024	0.001	<0.001	0.002	0.971	<0.001	<0.001	<0.001	0.464	0.968	0.046	0.274	<0.001	<0.001	0.031
Starch (%dm)	-0.129	1.000	-0.161	0.092	-0.189	0.148	-0.399	-0.469	-0.369	-0.471	-0.248	-0.309	0.386	0.651	0.047	0.302	-0.176	-0.266	0.337	-0.026	0.100	0.458	0.620	0.292	-0.060	-0.181	-0.291	-0.209
	0.453	.	0.349	0.594	0.270	0.389	0.016	0.004	0.027	0.004	0.144	0.067	0.020	<0.001	0.787	0.074	0.304	0.117	0.044	0.879	0.561	0.005	<0.001	0.084	0.726	0.290	0.085	0.222
Protein (%dm)	0.404	-0.161	1.000	-0.255	-0.011	0.225	-0.397	-0.206	0.094	0.444	0.195	0.359	-0.006	-0.636	-0.153	0.066	0.197	0.369	-0.038	0.344	0.230	-0.138	-0.161	0.319	-0.591	0.635	-0.176	-0.574
	0.015	0.349	.	0.134	0.948	0.188	0.017	0.228	0.587	0.007	0.255	0.031	0.970	<0.001	0.374	0.704	0.250	0.027	0.827	0.040	0.177	0.421	0.347	0.058	<0.001	<0.001	0.304	<0.001
Fat (%dm)	-0.810	0.092	-0.255	1.000	-0.793	0.731	0.097	0.343	0.318	-0.676	-0.170	-0.754	0.747	0.358	-0.542	-0.499	0.579	-0.133	0.694	-0.810	-0.597	-0.345	-0.074	-0.063	0.376	-0.509	-0.655	0.166
	<0.001	0.594	0.134	.	<0.001	<0.001	0.574	0.041	0.059	<0.001	0.323	<0.001	<0.001	0.032	<0.001	0.002	<0.001	0.438	<0.001	<0.001	<0.001	0.039	0.669	0.716	0.024	0.002	<0.001	0.332
b-glucan (%dm)	0.772	-0.189	-0.011	-0.793	1.000	-0.729	0.229	-0.242	-0.136	0.714	0.258	0.702	-0.857	-0.122	0.498	0.491	-0.458	-0.148	-0.836	0.693	0.407	0.104	-0.120	0.153	-0.014	0.432	0.831	-0.028
	<0.001	0.270	0.948	<0.001	.	<0.001	0.178	0.156	0.428	<0.001	0.129	<0.001	<0.001	0.479	0.002	0.002	0.005	0.390	<0.001	<0.001	0.014	0.546	0.487	0.372	0.935	0.009	<0.001	0.869
Mg (mg/kg dm)	-0.684	0.148	0.225	0.731	-0.729	1.000	0.007	0.313	0.555	-0.529	0.080	-0.518	0.686	0.124	-0.705	-0.425	0.800	0.175	0.680	-0.662	-0.595	-0.396	-0.125	0.114	0.011	-0.153	-0.765	-0.091
	<0.001	0.389	0.188	<0.001	<0.001	.	0.970	0.063	<0.001	<0.001	0.642	0.001	<0.001	0.472	<0.001	0.010	<0.001	0.307	<0.001	<0.001	<0.001	0.017	0.469	0.508	0.950	0.373	<0.001	0.596
K (mg/kg dm)	-0.077	-0.399	-0.397	0.097	0.229	0.007	1.000	0.108	0.515	0.118	0.330	0.037	-0.279	0.148	0.047	0.044	0.369	-0.037	-0.204	-0.202	-0.389	-0.357	-0.245	-0.127	0.698	0.047	0.289	0.333
	0.655	0.016	0.017	0.574	0.178	0.970	.	0.532	0.001	0.492	0.049	0.832	0.100	0.390	0.787	0.799	0.027	0.829	0.232	0.238	0.019	0.033	0.149	0.459	<0.001	0.787	0.087	0.047
Ca (mg/kg dm)	-0.598	-0.469	-0.206	0.343	-0.242	0.313	0.108	1.000	0.511	-0.256	0.171	-0.121	0.136	-0.100	-0.443	-0.731	0.303	0.160	0.153	-0.432	-0.389	-0.289	-0.524	-0.530	-0.119	-0.385	-0.114	0.479
	<0.001	0.004	0.228	0.041	0.156	0.063	0.532	.	0.001	0.131	0.318	0.482	0.430	0.563	0.007	<0.001	0.072	0.350	0.373	0.009	0.019	0.087	0.001	<0.001	0.490	0.020	0.510	0.003
P (mg/kg dm)	-0.319	-0.369	0.094	0.318	-0.136	0.555	0.515	0.511	1.000	-0.043	0.668	0.088	0.129	-0.050	-0.651	-0.505	0.619	0.288	0.061	-0.247	-0.330	-0.337	-0.377	-0.221	0.209	0.133	-0.053	0.206
	0.058	0.027	0.587	0.059	0.428	<0.001	0.001	0.001	.	0.801	<0.001	0.609	0.453	0.773	<0.001	0.002	<0.001	0.088	0.725	0.147	0.049	0.044	0.024	0.196	0.221	0.440	0.759	0.228
Diastatic power (°WK)	0.835	-0.471	0.444	-0.676	0.714	-0.529	0.118	-0.256	-0.043	1.000	0.250	0.754	-0.647	-0.508	0.307	0.445	-0.321	0.208	-0.766	0.596	0.277	-0.115	-0.292	0.177	-0.198	0.642	0.635	-0.211
	<0.001	0.004	0.007	<0.001	<0.001	<0.001	0.492	0.131	0.801	.	0.141	<0.001	<0.001	0.002	0.069	0.007	0.056	0.223	<0.001	<0.001	0.102	0.503	0.084	0.303	0.248	<0.001	<0.001	0.216
Alpha-amylase (CU/g dm)	0.197	-0.248	0.195	-0.170	0.258	0.080	0.330	0.171	0.668	0.250	1.000	0.334	-0.348	-0.070	-0.089	-0.063	0.214	0.227	-0.099	0.342	0.140	-0.173	-0.440	-0.187	0.056	0.438	0.265	0.245
	0.250	0.144	0.255	0.323	0.129	0.642	0.049	0.318	<0.001	0.141	.	0.047	0.038	0.684	0.607	0.716	0.211	0.183	0.565	0.041	0.417	0.314	0.007	0.274	0.747	0.008	0.118	0.150
Beta-amylase (B3U/g dm)	0.714	-0.309	0.359	-0.754	0.702	-0.518	0.037	-0.121	0.088	0.754	0.334	1.000	-0.582	-0.441	0.159	0.178	-0.271	0.235	-0.789	0.708	0.468	0.259	-0.023	0.082	-0.378	0.710	0.700	-0.331
	<0.001	0.067	0.031	<0.001	<0.001	0.001	0.832	0.482	0.609	<0.001	0.047	.	<0.001	0.007	0.356	0.300	0.110	0.167	<0.001	<0.001	0.004	0.127	0.894	0.635	0.023	<0.001	<0.001	0.049
Gelatinization T (°C)	-0.712	0.386	-0.006	0.747	-0.857	0.686	-0.279	0.136	0.129	-0.647	-0.348	-0.582	1.000	0.355	-0.555	-0.425	0.308	0.086	0.686	-0.619	-0.307	0.131	0.307	-0.149	0.023	-0.410	-0.750	-0.055
	<0.001	0.020	0.970	<0.001	<0.001	<0.001	0.100	0.430	0.453	<0.001	0.038	<0.001	.	0.034	<0.001	0.010	0.068	0.619	<0.001	<0.001	0.069	0.446	0.068	0.387	0.892	0.013	<0.001	0.750
C-Saccharification time (min)	-0.377	0.651	-0.636	0.358	-0.122	0.124	0.148	-0.100	-0.050	-0.508	-0.070	-0.441	0.355	1.000	0.096	0.081	-0.052	-0.451	0.262	-0.240	-0.144	0.225	0.255	-0.050	0.427	-0.457	-0.114	0.317
	0.024	<0.001	<0.001	0.032	0.479	0.472	0.390	0.563	0.773	0.002	0.684	0.007	0.034	.	0.577	0.638	0.764	0.006	0.123	0.159	0.402	0.187	0.134	0.774	0.009	0.005	0.508	0.060
C-Filtrate60 (g)	0.513	0.047	-0.153	-0.542	0.498	-0.705	0.047	-0.443	-0.651	0.307	-0.089	0.159	-0.555	0.096	1.000	0.626	-0.441	-0.263	-0.249	0.500	0.358	0.142	0.006	0.072	0.031	0.181	0.390	0.038
	0.001	0.787	0.374	<0.001	0.002	<0.001	0.787	0.007	<0.001	0.069	0.607	0.356	<0.001	0.577	.	<0.001	0.007	0.122	0.143	0.002	0.032	0.409	0.974	0.678	0.860	0.290	0.019	0.826
C-Extract content (°P)	0.634	0.302	0.066	-0.499	0.491	-0.425	0.044	-0.731	-0.505	0.445	-0.063	0.178	-0.425	0.081	0.626	1.000	-0.355	-0.059	-0.242	0.367	0.141	0.029	0.144	0.414	0.067	0.379	0.246	-0.283
	<0.001	0.074	0.704	0.002	0.002	0.010	0.799	<0.001	0.002	0.007	0.716	0.300	0.010	0.638	<0.001	.	0.034	0.731	0.154	0.028	0.411	0.868	0.401	0.012	0.699	0.022	0.148	0.095
C-pH	-0.502	-0.176	0.197	0.579	-0.458	0.800	0.369	0.303	0.619	-0.321	0.214	-0.271	0.308	-0.052	-0.441	-0.355	1.000	0.089	0.453	-0.528	-0.610	-0.574	-0.291	0.206	0.139	0.133	-0.475	-0.176
	0.002	0.304	0.250	<0.001	0.005	<0.001	0.027	0.072	<0.001	0.056	0.211	0.110	0.068	0.764	0.007	0.034	.	0.606	0.005	<0.001	<0.001	<0.001	0.085	0.229	0.418	0.439	0.003	0.304
C-FAN (mg/L)	0.006	-0.266	0.369	-0.133	-0.148	0.175	-0.037	0.160	0.288	0.208	0.227	0.235	0.086	-0.451	-0.263	-0.059	0.089	1.000	0.072	0.039	-0.021	0.067	-0.007	-0.247	-0.292	0.322	-0.213	-0.012
	0.971	0.117	0.027	0.438	0.390	0.307	0.829	0.350	0.088	0.223	0.183	0.167	0.619	0.006	0.122	0.731	0.606	.	0.676	0.821	0.902	0.700	0.968	0.146	0.084	0.056	0.211	0.942
C-Glucose (g/L)	-0.691	0.337	-0.038	0.694	-0.836	0.680	-0.204	0.153	0.061	-0.766	-0.099	-0.789	0.686	0.262	-0.249	-0.242	0.453	0.072	1.000	-0.593	-0.364	-0.171	0.027	-0.161	0.086	-0.347	-0.835	0.063
	<0.001	0.044	0.827	<0.001	<0.001	<0.001	0.232	0.373	0.725	<0.001	0.565	<0.001	<0.001	0.123	0.143	0.154	0.005	0.676	.	<0.001	0.029	0.319	0.876	0.348	0.617	0.038	<0.001	0.716
C-Maltose (g/L)	0.822	-0.026	0.344	-0.810	0.693	-0.662	-0.202	-0.432	-0.247	0.596	0.342	0.708	-0.619	-0.240	0.500	0.367	-0.528	0.039	-0.593	1.000	0.875	0.466	0.137	0.116	-0.361	0.601	0.589	-0.185
	<0.001	0.879	0.040	<0.001	<0.001	<0.001	0.238	0.009	0.147	<0.001	0.041	<0.001	<0.001	0.159	0.002	0.028	<0.001	0.821	<0.001	.	<0.001	0.004	0.427	0.499	0.031	<0.001	<0.001	0.280
C-Maltotriose (g/L)	0.579	0.100	0.230	-0.597	0.407	-0.595	-0.389	-0.389	-0.330	0.277	0.140	0.468	-0.307	-0.144	0.358	0.141	-0.610	-0.021	-0.364	0.875	1.000	0.688	0.376	-0.063	-0.318	0.314	0.378	-0.136
	<0.001	0.561	0.177	<0.001	0.014	<0.001	0.019	0.019	0.049	0.102	0.417	0.004	0.069	0.402	0.032	0.411	<0.001	0.902	0.029	<0.001	.	<0.001	0.024	0.716	0.059	0.062	0.023	0.429
C-Maltotetraose (g/L)	0.126	0.458	-0.138	-0.345	0.104	-0.396	-0.357	-0.289	-0.337	-0.115	-0.173	0.259	0.131	0.225	0.142	0.029	-0.574	0.067	-0.171	0.466	0.688	1.000	0.790	-0.205	-0.289	0.012	0.180	-0.041
	0.464	0.005	0.421	0.039	0.546	0.017	0.033	0.087	0.044	0.503	0.314	0.127	0.446	0.187	0.409	0.868	<0.001	0.700	0.319	0.004	<0.001	.	<0.001	0.230	0.087	0.944	0.294	0.811
C-Maltopentaose (g/L)	-0.007	0.620	-0.161	-0.074	-0.120	-0.125	-0.245	-0.524	-0.377	-0.292	-0.440	-0.023	0.307	0.255	0.006	0.144	-0.291	-0.007	0.027	0.137	0.376	0.790	1.000	0.154	-0.049	-0.042	-0.072	-0.317
	0.968	<0.001	0.347	0.669	0.487	0.469	0.149	0.001	0.024	0.084	0.007	0.894	0.068	0.134	0.974	0.401	0.085	0.968	0.876	0.427	0.024	<0.001	.	0.371	0.777	0.808	0.676	0.060
C-Viscosity (mPas)	0.334	0.292	0.319	-0.063	0.153	0.114	-0.127	-0.530	-0.221	0.177	-0.187	0.082	-0.149	-0.050	0.072	0.414	0.206	-0.247	-0.161	0.116	-0.063	-0.205	0.154	1.000	-0.132	0.370	0.003	-0.627
	0.046	0.084	0.058	0.716	0.372	0.508	0.459	<0.001	0.196	0.303	0.274	0.635	0.387	0.774	0.678	0.012	0.229	0.146	0.348	0.499	0.716	0.230	0.371	.	0.442	0.026	0.987	<0.001
C-Polyphenols (mg/L)	-0.187	-0.060	-0.591	0.376	-0.014	0.011	0.698	-0.119	0.209	-0.198	0.056	-0.378	0.023	0.427	0.031	0.067	0.139	-0.292	0.086	-0.361	-0.318	-0.289	-0.049	-0.132	1.000	-0.325	0.049	0.353
	0.274	0.726	<0.001	0.024	0.935	0.950	<0.001	0.490	0.221	0.248	0.747	0.023	0.892	0.009	0.860	0.699	0.418	0.084	0.617	0.031	0.059	0.087	0.777	0.442	.	0.053	0.775	0.035
C-Oplosbaar eiwit (g/L)	0.666	-0.181	0.635	-0.509	0.432	-0.153	0.047	-0.385	0.133	0.642	0.438	0.710	-0.410	-0.457	0.181	0.379	0.133	0.322	-0.347	0.601	0.314	0.012	-0.042	0.370	-0.325	1.000	0.325	-0.566
	<0.001	0.290	<0.001	0.002	0.009	0.373	0.787	0.020	0.440	<0.001	0.008	<0.001	0.013	0.005	0.290	0.022	0.439	0.056	0.038	<0.001	0.062	0.944	0.808	0.026	0.053	.	0.053	<0.001
C-Beta-glucan (mg/L)	0.639	-0.291	-0.176	-0.655	0.831	-0.765	0.289	-0.114	-0.053	0.635	0.265	0.700	-0.750	-0.114	0.390	0.246	-0.475	-0.213	-0.835	0.589	0.378	0.180	-0.072	0.003	0.049	0.325	1.000	0.056
	<0.001	0.085	0.304	<0.001	<0.001	<0.001	0.087	0.510	0.759	<0.001	0.118	<0.001	<0.001	0.508	0.019	0.148	0.003	0.211	<0.001	<0.001	0.023	0.294	0.676	0.987	0.775	0.053	.	0.747
C-Color	-0.359	-0.209	-0.574	0.166	-0.028	-0.091	0.333	0.479	0.206	-0.211	0.245	-0.331	-0.055	0.317	0.038	-0.283	-0.176	-0.012	0.063	-0.185	-0.136	-0.041	-0.317	-0.627	0.353	-0.566	0.056	1.000
	0.031	0.222	<0.001	0.332	0.869	0.596	0.047	0.003	0.228	0.216	0.150	0.049	0.750	0.060	0.826	0.095	0.304	0.942	0.716	0.280	0.429	0.811	0.060	<0.001	0.035	<0.001	0.747	.

Table S2: Bivariate Spearman correlation coefficients with their corresponding two-tailed significances between characteristics of 12 unmalted adjuncts (barley, wheat, einkorn, emmer, spelt, khorasan, quinoa, amaranth, buckwheat, sorghum, teff and tritordeum) [thousand kernel weight (TKW, g), starch (% dm), protein (% dm), fat (% dm), beta-glucan (% dm), magnesium (Mg, mg/kg dm), potassium (K, mg/kg dm), calcium (Ca, mg/kg dm), phosphorus (P, mg/kg dm), diastatic power (°WK), alpha-amylase (CU/g dm), beta-amylase (B3U/g dm) and gelatinization temperature (°C)], the Congress mashing process characteristics with 60% barley malt and 40% pregelatinized unmalted adjunct [saccharification time (min) and filtrate mass after 60 min (g)] and the resulting wort characteristics [extract content (°P), pH, free amino nitrogen (FAN, mg/L), glucose (g/L), maltose (g/L), maltotriose (g/L), maltotetraose (g/L) and maltopentaose (g/L)].

	TKW (g dm)	Starch (% dm)	Protein (% dm)	Fat (% dm)	Beta-glucan (% dm)	Mg (mg/kg dm)	K (mg/kg dm)	Ca (mg/kg dm)	P (mg/kg dm)	Diastatic power (°WK)	Alpha-amylase (CU/g dm)	Beta-amylase (B3U/g dm)	Gelatinization T (°C)	C-Saccharification time (min)	C-Filtrate mass after 60 min (g)	C-Extract content (°P)	C-pH	C-FAN (mg/L)	C-Glucose (g/L)	C-Maltose (g/L)	C-Maltotriose (g/L)	C-Maltotetraose (g/L)	C-Maltopentaose (g/L)
TKW (g dm)	1.000	-0.129	0.404	-0.810	0.772	-0.684	-0.077	-0.598	-0.319	0.835	0.197	0.714	-0.712	-0.376	0.377	-0.058	-0.422	-0.308	-0.166	0.052	0.209	0.140	-0.030
	.	0.453	0.015	<0.001	<0.001	<0.001	0.655	<0.001	0.058	<0.001	0.250	<0.001	<0.001	0.024	0.023	0.737	0.010	0.068	0.332	0.762	0.221	0.415	0.860
Starch (%dm)	-0.129	1.000	-0.161	0.092	-0.189	0.148	-0.399	-0.469	-0.369	-0.471	-0.248	-0.309	0.386	0.372	0.492	0.472	0.362	0.165	-0.146	0.311	0.239	0.181	0.097
	0.453	.	0.349	0.594	0.270	0.389	0.016	0.004	0.027	0.004	0.144	0.067	0.020	0.025	0.002	0.004	0.030	0.335	0.395	0.065	0.161	0.290	0.575
Protein (%dm)	0.404	-0.161	1.000	-0.255	-0.011	0.225	-0.397	-0.206	0.094	0.444	0.195	0.359	-0.006	-0.560	0.232	-0.098	0.193	-0.226	-0.176	-0.049	-0.065	-0.080	-0.321
	0.015	0.349	.	0.134	0.948	0.188	0.017	0.228	0.587	0.007	0.255	0.031	0.970	<0.001	0.174	0.568	0.261	0.185	0.304	0.778	0.705	0.645	0.057
Fat (%dm)	-0.810	0.092	-0.255	1.000	-0.793	0.731	0.097	0.343	0.318	-0.676	-0.170	-0.754	0.747	0.339	-0.141	0.101	0.424	0.007	0.287	0.053	-0.051	-0.014	0.187
	<0.001	0.594	0.134	.	<0.001	<0.001	0.574	0.041	0.059	<0.001	0.323	<0.001	<0.001	0.043	0.414	0.560	0.010	0.969	0.090	0.759	0.768	0.936	0.275
b-glucan (%dm)	0.772	-0.189	-0.011	-0.793	1.000	-0.729	0.229	-0.242	-0.136	0.714	0.258	0.702	-0.857	-0.085	0.092	0.056	-0.498	-0.054	-0.197	-0.082	0.085	0.119	0.144
	<0.001	0.270	0.948	<0.001	.	<0.001	0.178	0.156	0.428	<0.001	0.129	<0.001	<0.001	0.623	0.593	0.746	0.002	0.753	0.249	0.635	0.623	0.488	0.402
Mg (mg/kg dm)	-0.684	0.148	0.225	0.731	-0.729	1.000	0.007	0.313	0.555	-0.529	0.080	-0.518	0.686	0.105	-0.071	0.148	0.708	0.027	-0.146	-0.206	-0.374	-0.253	-0.051
	<0.001	0.389	0.188	<0.001	<0.001	.	0.970	0.063	<0.001	<0.001	0.642	0.001	<0.001	0.541	0.680	0.389	<0.001	0.874	0.395	0.229	0.025	0.137	0.767
K (mg/kg dm)	-0.077	-0.399	-0.397	0.097	0.229	0.007	1.000	0.108	0.515	0.118	0.330	0.037	-0.279	0.218	-0.191	-0.096	-0.064	0.037	-0.232	-0.617	-0.504	-0.283	0.249
	0.655	0.016	0.017	0.574	0.178	0.970	.	0.532	0.001	0.492	0.049	0.832	0.100	0.202	0.265	0.578	0.713	0.832	0.173	<0.001	0.002	0.095	0.143
Ca (mg/kg dm)	-0.598	-0.469	-0.206	0.343	-0.242	0.313	0.108	1.000	0.511	-0.256	0.171	-0.121	0.136	0.105	-0.626	-0.103	-0.004	0.213	0.249	-0.172	-0.293	-0.258	-0.107
	<0.001	0.004	0.228	0.041	0.156	0.063	0.532	.	0.001	0.131	0.318	0.482	0.430	0.543	<0.001	0.550	0.982	0.213	0.143	0.317	0.083	0.128	0.534
P (mg/kg dm)	-0.319	-0.369	0.094	0.318	-0.136	0.555	0.515	0.511	1.000	-0.043	0.668	0.088	0.129	0.100	-0.261	0.013	0.110	0.162	-0.336	-0.682	-0.750	-0.639	-0.158
	0.058	0.027	0.587	0.059	0.428	<0.001	0.001	0.001	.	0.801	<0.001	0.609	0.453	0.562	0.125	0.938	0.523	0.345	0.045	<0.001	<0.001	<0.001	0.356
Diastatic power (°WK)	0.835	-0.471	0.444	-0.676	0.714	-0.529	0.118	-0.256	-0.043	1.000	0.250	0.754	-0.647	-0.277	0.173	0.011	-0.437	-0.348	-0.041	-0.130	0.027	0.167	0.138
	<0.001	0.004	0.007	<0.001	<0.001	<0.001	0.492	0.131	0.801	.	0.141	<0.001	<0.001	0.102	0.313	0.948	0.008	0.038	0.811	0.450	0.875	0.331	0.424
Alpha-amylase (CU/g dm)	0.197	-0.248	0.195	-0.170	0.258	0.080	0.330	0.171	0.668	0.250	1.000	0.334	-0.348	-0.068	0.005	-0.044	-0.002	0.204	-0.482	-0.771	-0.750	-0.717	-0.281
	0.250	0.144	0.255	0.323	0.129	0.642	0.049	0.318	<0.001	0.141	.	0.047	0.038	0.695	0.975	0.798	0.992	0.232	0.003	<0.001	<0.001	<0.001	0.097
Beta-amylase (B3U/g dm)	0.714	-0.309	0.359	-0.754	0.702	-0.518	0.037	-0.121	0.088	0.754	0.334	1.000	-0.582	-0.262	0.142	0.096	-0.422	-0.015	-0.185	-0.153	-0.071	-0.077	-0.157
	<0.001	0.067	0.031	<0.001	<0.001	0.001	0.832	0.482	0.609	<0.001	0.047	.	<0.001	0.123	0.408	0.577	0.010	0.932	0.280	0.374	0.680	0.657	0.359
Gelatinization T (°C)	-0.712	0.386	-0.006	0.747	-0.857	0.686	-0.279	0.136	0.129	-0.647	-0.348	-0.582	1.000	0.395	0.131	0.306	0.382	0.135	0.251	0.185	0.039	0.098	0.105
	<0.001	0.020	0.970	<0.001	<0.001	<0.001	0.100	0.430	0.453	<0.001	0.038	<0.001	.	0.017	0.448	0.070	0.022	0.434	0.140	0.279	0.821	0.570	0.540
C-Saccharification time (min)	-0.376	0.372	-0.560	0.339	-0.085	0.105	0.218	0.105	0.100	-0.277	-0.068	-0.262	0.395	1.000	0.249	0.740	-0.024	0.421	0.177	-0.029	0.001	0.304	0.689
	0.024	0.025	<0.001	0.043	0.623	0.541	0.202	0.543	0.562	0.102	0.695	0.123	0.017	.	0.142	<0.001	0.889	0.010	0.300	0.866	0.996	0.071	<0.001
C-Filtrate60 (g)	0.377	0.492	0.232	-0.141	0.092	-0.071	-0.191	-0.626	-0.261	0.173	0.005	0.142	0.131	0.249	1.000	0.549	0.156	-0.126	0.069	0.151	0.199	0.347	0.364
	0.023	0.002	0.174	0.414	0.593	0.680	0.265	<0.001	0.125	0.313	0.975	0.408	0.448	0.142	.	<0.001	0.364	0.464	0.689	0.379	0.244	0.038	0.029
C-Extract content (°P)	-0.058	0.472	-0.098	0.101	0.056	0.148	-0.096	-0.103	0.013	0.011	-0.044	0.096	0.306	0.740	0.549	1.000	0.173	0.188	0.136	0.111	0.112	0.440	0.647
	0.737	0.004	0.568	0.560	0.746	0.389	0.578	0.550	0.938	0.948	0.798	0.577	0.070	<0.001	<0.001	.	0.312	0.273	0.429	0.518	0.514	0.007	<0.001
C-pH	-0.422	0.362	0.193	0.424	-0.498	0.708	-0.064	-0.004	0.110	-0.437	-0.002	-0.422	0.382	-0.024	0.156	0.173	1.000	-0.033	-0.147	-0.141	-0.270	-0.114	0.017
	0.010	0.030	0.261	0.010	0.002	<0.001	0.713	0.982	0.523	0.008	0.992	0.010	0.022	0.889	0.364	0.312	.	0.850	0.392	0.410	0.111	0.509	0.920
C-FAN (mg/L)	-0.308	0.165	-0.226	0.007	-0.054	0.027	0.037	0.213	0.162	-0.348	0.204	-0.015	0.135	0.421	-0.126	0.188	-0.033	1.000	-0.291	-0.314	-0.288	-0.244	-0.073
	0.068	0.335	0.185	0.969	0.753	0.874	0.832	0.213	0.345	0.038	0.232	0.932	0.434	0.010	0.464	0.273	0.850	.	0.085	0.062	0.089	0.151	0.673
C-Glucose (g/L)	-0.166	-0.146	-0.176	0.287	-0.197	-0.146	-0.232	0.249	-0.336	-0.041	-0.482	-0.185	0.251	0.177	0.069	0.136	-0.147	-0.291	1.000	0.602	0.604	0.584	0.392
	0.332	0.395	0.304	0.090	0.249	0.395	0.173	0.143	0.045	0.811	0.003	0.280	0.140	0.300	0.689	0.429	0.392	0.085	.	<0.001	<0.001	<0.001	0.018
C-Maltose (g/L)	0.052	0.311	-0.049	0.053	-0.082	-0.206	-0.617	-0.172	-0.682	-0.130	-0.771	-0.153	0.185	-0.029	0.151	0.111	-0.141	-0.314	0.602	1.000	0.949	0.692	0.172
	0.762	0.065	0.778	0.759	0.635	0.229	<0.001	0.317	<0.001	0.450	<0.001	0.374	0.279	0.866	0.379	0.518	0.410	0.062	<0.001	.	<0.001	<0.001	0.316
C-Maltotriose (g/L)	0.209	0.239	-0.065	-0.051	0.085	-0.374	-0.504	-0.293	-0.750	0.027	-0.750	-0.071	0.039	0.001	0.199	0.112	-0.270	-0.288	0.604	0.949	1.000	0.782	0.286
	0.221	0.161	0.705	0.768	0.623	0.025	0.002	0.083	<0.001	0.875	<0.001	0.680	0.821	0.996	0.244	0.514	0.111	0.089	<0.001	<0.001	.	<0.001	0.090
C-Maltotetraose (g/L)	0.140	0.181	-0.080	-0.014	0.119	-0.253	-0.283	-0.258	-0.639	0.167	-0.717	-0.077	0.098	0.304	0.347	0.440	-0.114	-0.244	0.584	0.692	0.782	1.000	0.713
	0.415	0.290	0.645	0.936	0.488	0.137	0.095	0.128	<0.001	0.331	<0.001	0.657	0.570	0.071	0.038	0.007	0.509	0.151	<0.001	<0.001	<0.001	.	<0.001
C-Maltopentaose (g/L)	-0.030	0.097	-0.321	0.187	0.144	-0.051	0.249	-0.107	-0.158	0.138	-0.281	-0.157	0.105	0.689	0.364	0.647	0.017	-0.073	0.392	0.172	0.286	0.713	1.000
	0.860	0.575	0.057	0.275	0.402	0.767	0.143	0.534	0.356	0.424	0.097	0.359	0.540	<0.001	0.029	<0.001	0.920	0.673	0.018	0.316	0.090	<0.001	.

Table S3: Bivariate Spearman correlation coefficients with their corresponding two-tailed significances between characteristics of 12 unmalted adjuncts (barley, wheat, einkorn, emmer, spelt, khorasan, quinoa, amaranth, buckwheat, sorghum, teff and tritordeum) [thousand kernel weight (TKW, g), starch (% dm), protein (% dm), fat (% dm), beta-glucan (% dm), magnesium (Mg, mg/kg dm), potassium (K, mg/kg dm), calcium (Ca, mg/kg dm), phosphorus (P, mg/kg dm), diastatic power (°WK), alpha-amylase (CU/g dm), beta-amylase (B3U/g dm) and gelatinization temperature (°C)], the Evans mashing process characteristics with 60% barley malt and 40% unmalted adjunct [saccharification time (min) and filtrate mass after 60 min (g)] and the resulting wort characteristics [extract content (°P), pH, free amino nitrogen (FAN, mg/L), glucose (g/L), maltose (g/L), maltotriose (g/L), maltotetraose (g/L) and maltopentaose (g/L)].

	TKW (g dm)	Starch (% dm)	Protein (% dm)	Fat (% dm)	Beta-glucan (% dm)	Mg (mg/kg dm)	K (mg/kg dm)	Ca (mg/kg dm)	P (mg/kg dm)	Diastatic power (°WK)	Alpha-amylase (CU/g dm)	Beta-amylase (B3U/g dm)	Gelatinization T (°C)	C-Saccharification time (min)	C-Filtrate mass after 60 min (g)	C-Extract content (°P)	C-pH	C-FAN (mg/L)	C-Glucose (g/L)	C-Maltose (g/L)	C-Maltotriose (g/L)	C-Maltotetraose (g/L)	C-Maltopentaose (g/L)
TKW (g dm)	1.000	-0.129	0.404	-0.810	0.772	-0.684	-0.077	-0.598	-0.319	0.835	0.197	0.714	-0.712	0.064	-0.149	0.667	-0.165	0.098	-0.250	0.650	0.658	0.484	0.248
	.	0.453	0.015	<0.001	<0.001	<0.001	0.655	<0.001	0.058	<0.001	0.250	<0.001	<0.001	0.713	0.384	<0.001	0.335	0.569	0.142	<0.001	<0.001	0.003	0.145
Starch (%dm)	-0.129	1.000	-0.161	0.092	-0.189	0.148	-0.399	-0.469	-0.369	-0.471	-0.248	-0.309	0.386	0.197	0.055	0.159	-0.228	-0.222	-0.200	-0.326	-0.334	0.014	0.266
	0.453	.	0.349	0.594	0.270	0.389	0.016	0.004	0.027	0.004	0.144	0.067	0.020	0.251	0.748	0.356	0.181	0.194	0.243	0.052	0.047	0.937	0.117
Protein (%dm)	0.404	-0.161	1.000	-0.255	-0.011	0.225	-0.397	-0.206	0.094	0.444	0.195	0.359	-0.006	0.047	-0.008	0.271	0.454	-0.282	-0.151	-0.015	-0.045	-0.021	0.036
	0.015	0.349	.	0.134	0.948	0.188	0.017	0.228	0.587	0.007	0.255	0.031	0.970	0.784	0.961	0.110	0.005	0.096	0.381	0.929	0.796	0.902	0.836
Fat (%dm)	-0.810	0.092	-0.255	1.000	-0.793	0.731	0.097	0.343	0.318	-0.676	-0.170	-0.754	0.747	0.334	0.325	-0.387	0.347	-0.133	0.425	-0.736	-0.727	-0.506	-0.313
	<0.001	0.594	0.134	.	<0.001	<0.001	0.574	0.041	0.059	<0.001	0.323	<0.001	<0.001	0.046	0.053	0.020	0.038	0.438	0.010	<0.001	<0.001	0.002	0.063
b-glucan (%dm)	0.772	-0.189	-0.011	-0.793	1.000	-0.729	0.229	-0.242	-0.136	0.714	0.258	0.702	-0.857	-0.193	-0.283	0.508	-0.410	0.340	-0.268	0.771	0.765	0.470	0.220
	<0.001	0.270	0.948	<0.001	.	<0.001	0.178	0.156	0.428	<0.001	0.129	<0.001	<0.001	0.259	0.094	0.002	0.013	0.043	0.113	<0.001	<0.001	0.004	0.197
Mg (mg/kg dm)	-0.684	0.148	0.225	0.731	-0.729	1.000	0.007	0.313	0.555	-0.529	0.080	-0.518	0.686	0.127	0.019	-0.221	0.629	-0.159	0.203	-0.784	-0.825	-0.658	-0.376
	<0.001	0.389	0.188	<0.001	<0.001	.	0.970	0.063	<0.001	<0.001	0.642	0.001	<0.001	0.460	0.912	0.196	<0.001	0.355	0.235	<0.001	<0.001	<0.001	0.024
K (mg/kg dm)	-0.077	-0.399	-0.397	0.097	0.229	0.007	1.000	0.108	0.515	0.118	0.330	0.037	-0.279	-0.036	-0.320	0.210	0.227	0.698	0.188	-0.020	0.000	-0.133	-0.403
	0.655	0.016	0.017	0.574	0.178	0.970	.	0.532	0.001	0.492	0.049	0.832	0.100	0.833	0.057	0.219	0.183	<0.001	0.271	0.907	0.999	0.439	0.015
Ca (mg/kg dm)	-0.598	-0.469	-0.206	0.343	-0.242	0.313	0.108	1.000	0.511	-0.256	0.171	-0.121	0.136	-0.339	0.139	-0.679	0.023	-0.096	0.207	0.025	-0.012	-0.245	-0.202
	<0.001	0.004	0.228	0.041	0.156	0.063	0.532	.	0.001	0.131	0.318	0.482	0.430	0.043	0.419	<0.001	0.896	0.577	0.226	0.886	0.943	0.149	0.236
P (mg/kg dm)	-0.319	-0.369	0.094	0.318	-0.136	0.555	0.515	0.511	1.000	-0.043	0.668	0.088	0.129	-0.197	-0.135	-0.112	0.559	0.272	0.039	-0.141	-0.184	-0.394	-0.498
	0.058	0.027	0.587	0.059	0.428	<0.001	0.001	0.001	.	0.801	<0.001	0.609	0.453	0.251	0.432	0.516	<0.001	0.108	0.821	0.412	0.282	0.017	0.002
Diastatic power (°WK)	0.835	-0.471	0.444	-0.676	0.714	-0.529	0.118	-0.256	-0.043	1.000	0.250	0.754	-0.647	-0.029	-0.159	0.439	-0.056	0.051	-0.196	0.591	0.587	0.427	0.176
	<0.001	0.004	0.007	<0.001	<0.001	<0.001	0.492	0.131	0.801	.	0.141	<0.001	<0.001	0.868	0.356	0.007	0.746	0.767	0.252	<0.001	<0.001	0.009	0.303
Alpha-amylase (CU/g dm)	0.197	-0.248	0.195	-0.170	0.258	0.080	0.330	0.171	0.668	0.250	1.000	0.334	-0.348	-0.163	-0.153	0.254	0.414	0.291	-0.101	0.267	0.218	-0.194	-0.435
	0.250	0.144	0.255	0.323	0.129	0.642	0.049	0.318	<0.001	0.141	.	0.047	0.038	0.342	0.374	0.135	0.012	0.086	0.558	0.116	0.201	0.257	0.008
Beta-amylase (B3U/g dm)	0.714	-0.309	0.359	-0.754	0.702	-0.518	0.037	-0.121	0.088	0.754	0.334	1.000	-0.582	-0.318	-0.204	0.273	-0.176	0.147	-0.420	0.693	0.666	0.498	0.197
	<0.001	0.067	0.031	<0.001	<0.001	0.001	0.832	0.482	0.609	<0.001	0.047	.	<0.001	0.059	0.233	0.108	0.304	0.393	0.011	<0.001	<0.001	0.002	0.249
Gelatinization T (°C)	-0.712	0.386	-0.006	0.747	-0.857	0.686	-0.279	0.136	0.129	-0.647	-0.348	-0.582	1.000	0.254	0.434	-0.497	0.224	-0.421	0.071	-0.770	-0.756	-0.250	0.027
	<0.001	0.020	0.970	<0.001	<0.001	<0.001	0.100	0.430	0.453	<0.001	0.038	<0.001	.	0.134	0.008	0.002	0.188	0.011	0.683	<0.001	<0.001	0.141	0.877
C-Saccharification time (min)	0.064	0.197	0.047	0.334	-0.193	0.127	-0.036	-0.339	-0.197	-0.029	-0.163	-0.318	0.254	1.000	0.592	0.379	0.229	-0.209	0.636	-0.289	-0.269	-0.103	-0.087
	0.713	0.251	0.784	0.046	0.259	0.460	0.833	0.043	0.251	0.868	0.342	0.059	0.134	.	<0.001	0.023	0.179	0.221	<0.001	0.088	0.112	0.549	0.612
C-Filtrate60 (g)	-0.149	0.055	-0.008	0.325	-0.283	0.019	-0.320	0.139	-0.135	-0.159	-0.153	-0.204	0.434	0.592	1.000	-0.226	-0.148	-0.455	0.348	-0.067	-0.034	0.252	0.295
	0.384	0.748	0.961	0.053	0.094	0.912	0.057	0.419	0.432	0.356	0.374	0.233	0.008	<0.001	.	0.184	0.387	0.005	0.037	0.699	0.846	0.138	0.081
C-Extract content (°P)	0.667	0.159	0.271	-0.387	0.508	-0.221	0.210	-0.679	-0.112	0.439	0.254	0.273	-0.497	0.379	-0.226	1.000	0.191	0.345	0.105	0.201	0.196	0.083	-0.063
	<0.001	0.356	0.110	0.020	0.002	0.196	0.219	<0.001	0.516	0.007	0.135	0.108	0.002	0.023	0.184	.	0.264	0.040	0.541	0.239	0.252	0.631	0.713
C-pH	-0.165	-0.228	0.454	0.347	-0.410	0.629	0.227	0.023	0.559	-0.056	0.414	-0.176	0.224	0.229	-0.148	0.191	1.000	0.196	0.325	-0.423	-0.454	-0.660	-0.680
	0.335	0.181	0.005	0.038	0.013	<0.001	0.183	0.896	<0.001	0.746	0.012	0.304	0.188	0.179	0.387	0.264	.	0.252	0.053	0.010	0.005	<0.001	<0.001
C-FAN (mg/L)	0.098	-0.222	-0.282	-0.133	0.340	-0.159	0.698	-0.096	0.272	0.051	0.291	0.147	-0.421	-0.209	-0.455	0.345	0.196	1.000	0.082	0.201	0.208	-0.042	-0.320
	0.569	0.194	0.096	0.438	0.043	0.355	<0.001	0.577	0.108	0.767	0.086	0.393	0.011	0.221	0.005	0.040	0.252	.	0.636	0.240	0.224	0.807	0.057
C-Glucose (g/L)	-0.250	-0.200	-0.151	0.425	-0.268	0.203	0.188	0.207	0.039	-0.196	-0.101	-0.420	0.071	0.636	0.348	0.105	0.325	0.082	1.000	-0.082	-0.079	-0.281	-0.279
	0.142	0.243	0.381	0.010	0.113	0.235	0.271	0.226	0.821	0.252	0.558	0.011	0.683	<0.001	0.037	0.541	0.053	0.636	.	0.633	0.646	0.097	0.100
C-Maltose (g/L)	0.650	-0.326	-0.015	-0.736	0.771	-0.784	-0.020	0.025	-0.141	0.591	0.267	0.693	-0.770	-0.289	-0.067	0.201	-0.423	0.201	-0.082	1.000	0.992	0.553	0.287
	<0.001	0.052	0.929	<0.001	<0.001	<0.001	0.907	0.886	0.412	<0.001	0.116	<0.001	<0.001	0.088	0.699	0.239	0.010	0.240	0.633	.	<0.001	<0.001	0.089
C-Maltotriose (g/L)	0.658	-0.334	-0.045	-0.727	0.765	-0.825	0.000	-0.012	-0.184	0.587	0.218	0.666	-0.756	-0.269	-0.034	0.196	-0.454	0.208	-0.079	0.992	1.000	0.608	0.329
	<0.001	0.047	0.796	<0.001	<0.001	<0.001	0.999	0.943	0.282	<0.001	0.201	<0.001	<0.001	0.112	0.846	0.252	0.005	0.224	0.646	<0.001	.	<0.001	0.050
C-Maltotetraose (g/L)	0.484	0.014	-0.021	-0.506	0.470	-0.658	-0.133	-0.245	-0.394	0.427	-0.194	0.498	-0.250	-0.103	0.252	0.083	-0.660	-0.042	-0.281	0.553	0.608	1.000	0.844
	0.003	0.937	0.902	0.002	0.004	<0.001	0.439	0.149	0.017	0.009	0.257	0.002	0.141	0.549	0.138	0.631	<0.001	0.807	0.097	<0.001	<0.001	.	<0.001
C-Maltopentaose (g/L)	0.248	0.266	0.036	-0.313	0.220	-0.376	-0.403	-0.202	-0.498	0.176	-0.435	0.197	0.027	-0.087	0.295	-0.063	-0.680	-0.320	-0.279	0.287	0.329	0.844	1.000
	0.145	0.117	0.836	0.063	0.197	0.024	0.015	0.236	0.002	0.303	0.008	0.249	0.877	0.612	0.081	0.713	<0.001	0.057	0.100	0.089	0.050	<0.001	.
